# Reversing selectivity of bambusuril macrocycles toward inorganic anions by installing spacious substituents on their portals†

**DOI:** 10.1039/d4sc07150f

**Published:** 2024-12-06

**Authors:** Carola Rando, Surbhi Grewal, Jan Sokolov, Petr Kulhánek, Vladimír Šindelář

**Affiliations:** a Department of Chemistry, Faculty of Science, Masaryk University Kamenice 5 625 00 Brno Czech Republic; b RECETOX, Faculty of Science, Masaryk University Kamenice 5 625 00 Brno Czech Republic sindelar@chemi.muni.cz; c National Centre for Biomolecular Research, Faculty of Science, Masaryk University Kamenice 5 625 00 Brno Czech Republic kulhanek@chemi.muni.cz

## Abstract

Two chiral bambusurils, which are diastereomers to each other, show remarkable differences in their binding affinity and selectivity toward inorganic anions as determined by isothermal titration calorimetry. These differences are explained by quantum-chemical calculations.

## Abstract 2

The order of bulky 1-phenylethyl and small methyl substituents of the macrocycle building blocks has a major impact on the affinity and selectivity of the macrocycle toward inorganic anions.

## Graphical abstract

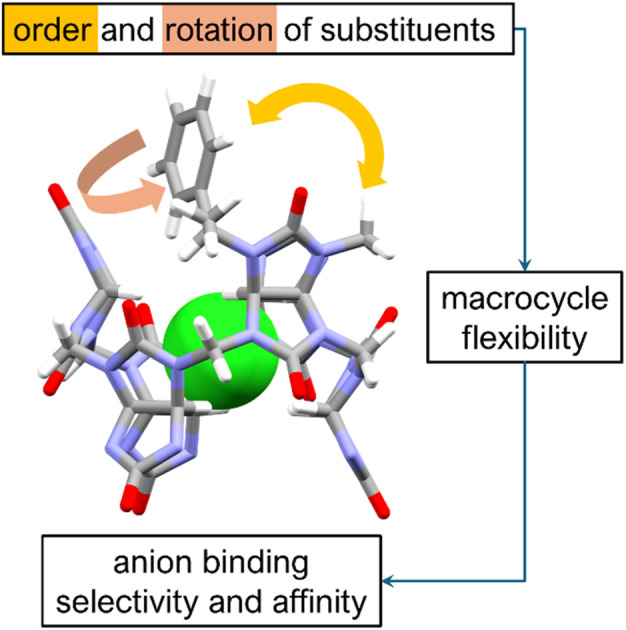

## Introduction

Artificial molecules with the ability to act as receptors of inorganic anions are investigated in many fields, including anion sensing,^1,2^ organocatalysis,^3,4^ anion remediation in industrial wastewater,^5–7^ and others. Supramolecular chemists dealing with the host–guest chemistry of inorganic anions are intrigued by the design of anion receptors with optimal binding affinity and selectivity toward the selected analyte.^3,7–9^ This design is particularly challenging in the case of receptors for inorganic anions featuring relatively small differences in their sizes. Among supramolecular host molecules, macrocycles are especially appreciated for their ability to discriminate particular anions because they are more rigid compared to their acyclic analogues.^10^ Macrocycles are valued for their high affinity, which is achieved thanks to multiple binding motifs. Moreover, the selectivity of macrocycles toward a particular anion can be tuned by the number of their repeating units, as can be illustrated on calix[*n*]pyrroles or hemicucurbit[*n*]urils. While calix[4]pyrroles show 100 times higher selectivity for fluoride compared to other halides and phosphates, calix[6]pyrroles preferentially bind iodide.^11^ Similarly, hemicucurbit[6]urils prefer to bind chloride over iodide, while hemicucurbit[8]urils form the most stable complexes with PF_6_^−^ and SbF_6_^−^.^12^

**Fig. 1:**
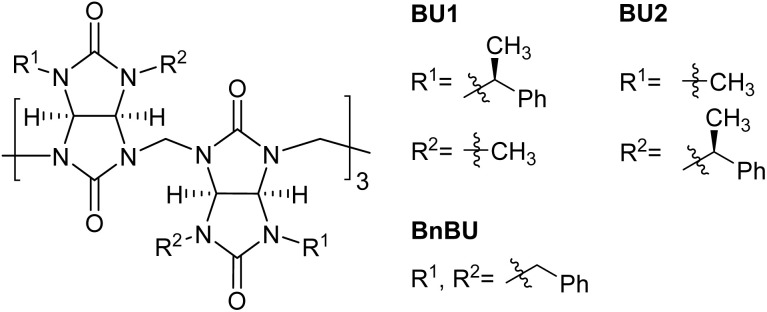
Structures of bambusuril macrocycles investigated in this work.

Bambus[*n*]urils are anion receptors consisting of *n* glycoluril units connected by *n* methylene bridges.^13,14^ Until now, only four (*n* = 4) and six (*n* = 6) membered bambusuril homologues have been reported.^15,16^ As the four-membered bambusurils do not bind to any anion, the six-membered homologues form stable complexes with many inorganic anions. The typical complex in the solution consists of a single anion included within the center of the macrocycle cavity. It has been reported that the binding affinity of bambus[6]urils (BU[6]s) to anions can be modulated by installing different substituents on the nitrogen atoms of the BU[6] portals.^17,18^ However, independently of their substituents, BU[6]s have shown essentially the same selectivity for all inorganic anions ranging from small F^−^ to large ClO_4_^−^ in the same solvent. This is mainly due to the flexibility of the BU[6] macrocyclic framework and its ability to adapt to the size of these anions. Thus, the selectivity of BU[6] is mostly dictated by anion solvation and correlates with the charge density of anions.^13,19^ Previously, we prepared enantiomerically pure bambusurils BU1 and BU2 (Fig. 1) and demonstrated their enantioselectivity in binding different chiral carboxylates.^20^ Here, we demonstrate that diastereomers BU1 and BU2 bind the same inorganic anions with very different affinities and selectivities. To the best of our knowledge, this is the first example of any receptor of inorganic anions, in which the type and position of substituents attached to the receptor binding site impact the receptor selectivity. The binding differences were rationalized with the help of theoretical calculations.

## Results and discussion

The binding of BU1 and BU2 with a series of inorganic anions was investigated by isothermal titration calorimetry in chloroform. For all titrations, the experimental data fit well with the 1 : 1 binding model, which is consistent with the 1 : 1 host–guest stoichiometry previously described for the number of bambusuril-anion complexes. The reported association constants are apparent, as we did not consider the competition of TBA cations and bambusuril macrocycles for anions.

**Fig. 2:**
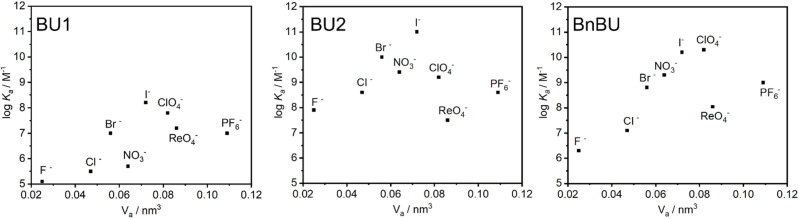
Dependence of apparent association constants on anions size.

When BU1 and BU2 were compared, striking differences in their binding affinities of more than two orders of magnitude were observed (Table 1). For example, the binding selectivity of BU2 over BU1 for F^−^, Cl^−^, Br^−^, and I^−^ is 592, 1273, 891, and 750. The highest selectivity of BU2 over BU1 of 5822 was observed for NO_3_^−^. We also compared the binding characteristics of BU1 and BU2 to those of the previously studied BnBU (Table 1 and Fig. 2).^19^ This macrocycle bears 12 benzyl substituents on its portals. We compared the binding affinities of all three bambusurils according to the volume of bound anions. The stability of complexes with BU1 and BU2 increases from F^−^ to I^−^, while it decreases for anions with large diameters, such as ClO_4_^−^, ReO_4_^−^, and PF_6_^−^. In contrast, the binding affinity of BnBU increases from F^−^ up to ClO_4_^−^ and decreases for the larger anions. This clearly illustrates that the cavity of BnBU is more flexible and can even adapt to larger ClO_4_^−^, while the BU1 and BU2 cavities are best suited for smaller I^−^ and bind ClO_4_^−^ less efficiently.

**Table 1:** Apparent association constants (*K*_a_, M^−1^) of BUs with various anions in CHCl_3_ at 298.15 K determined by ITC

Anions	BU1	BU2	BnBU^a^
CH_3_CO_2_^−^	1.9 × 10^4^	2.0 × 10^6^	5.6 × 10^5^
MeSO_3_^−^	8.2 × 10^4^	9.4 × 10^5^	7.3 × 10^5^
NO_3_^−^	5.1 × 10^5^	3.0 × 10^9^	1.8 × 10^9^
ReO_4_^−^	1.7 × 10^7^	3.0 × 10^7^	1.1 × 10^8^
ClO_4_^−^	6.8 × 10^7^	1.7 × 10^9^	2.1 × 10^10^
PF_6_^−^	1.2 × 10^7^	4.1 × 10^8^	8.7 × 10^8^
F^−^	1.3 × 10^5^	7.7 × 10^7^	1.9 × 10^6^
Cl^−^	3.3 × 10^5^	4.2 × 10^8^	1.3 × 10^7^
Br^−^	1.1 × 10^7^	9.8 × 10^9^	6.7 × 10^8^
I^−^	1.6 × 10^8^	1.2 × 10^11^	1.6 × 10^10^

a Published previously, see ref. 19. Standard deviations were calculated from two or three independent experiments and are lower than 17%, except for the BU2·ReO_4_^−^ complex, for which it is 30%.

The absolute values of association constants of BnBU complexes are comparable with those of BU2 but significantly higher than those of BU1 (Table 1). Further comparison of BnBU with BU2 revealed that all halides are bound by BU2 more strongly than by BnBU, but the reverse is observed for ClO_4_^−^ and large anions. This clearly shows the preferential binding of BU2 to smaller spherical anions.

Another crucial difference between the investigated bambusurils is in their selectivity. Specifically, BnBU preferentially binds ClO_4_^−^ over Cl^−^ with selectivity exceeding 1600. In contrast, the selectivity for ClO_4_^−^ over Cl^−^ in the cases of BU1 and BU2 is reduced to 206 and 4. Moreover, BnBU and BU1 bind ReO_4_^−^ more than 8 and 50 times more strongly than Cl^−^, while BU2 preferentially binds Cl^−^ over ReO_4_^−^ with a selectivity of 14. The reverse selectivity among the investigated bambusurils was observed also for the NO_3_^−^/Br^−^ anion pair (Table 1). Inspired by the reviewers' comments, we decided to test if the reverse selectivity will be recorded in a solvent different from chloroform. We determined the association constants for the complexes of BU1 and TBA salts of Br^−^ (2.6 × 10^7^ M^−1^) and NO_3_^−^ (2.9 × 10^6^ M^−1^) in acetonitrile (Table S3†). The results showed that BU1 preferentially binds Br^−^ over NO_3_^−^ in both chloroform and acetonitrile with selectivities of 22 and 9, while BnBU preferentially binds NO_3_^−^ over Br^−^ with a selectivity of 3 in chloroform.

These results clearly show that replacing benzyl with (*S*)-1-phenylethyl substituents in the bambusuril structure leads to changes in binding affinity and, consequently, to reverse selectivity toward anions. This contrasts with previous studies,^17,21^ in which the selectivity toward anions remained the same for differently substituted bambusurils.

We also decided to test whether the observed differences in anion binding could be rationalized by the ability of bambusuril to act as a heterotopic ion-pair receptor interacting simultaneously with the anion and cation of the salt. We conducted an ITC titration of BU1 with tetraethylammonium (TEA) chloride in chloroform. We determined that *K*_a_ for the BU1·Cl^−^ complex is about 4.6 times lower when TEA instead of TBA salt was used. This reflects the higher affinity of TEA towards Cl^−^ compared to TBA and supports that the bambusuril does not act as a heterotopic ion-pair receptor.

We also performed ^1^H NMR titration of the BU2 solution using TBACl in chloroform (Fig. S24†). The results showed that TBA proton signals experience an upfield chemical shift in the presence of BU2. This is explained by shielding TBA^+^ from the chloride anion encapsulated inside the macrocycle, excluding the possibility of BU2 cooperatively interacting with the cation. The binding of chloride within the BU2 cavity is slow on the NMR time scale, in contrast to TBA^+^, which exhibits fast exchange upon interaction with the macrocycle. This indicates that the TBACl ion pair dissociates upon interacting with the macrocycle. The DOSY spectra of an equimolar solution of TBACl and BU2 in chloroform showed that the diffusion coefficient for TBA^+^ is higher compared to that of the macrocycle but lower than that of TBA^+^ measured in the absence of the macrocycle (Fig. S25 and S26†). This indicates that TBA^+^ and the BU2·Cl^−^ complex interact to some extent. We performed dilution experiments of an equimolar mixture of BU2 and TBACl in chloroform to determine the ion-pair association constant (*K*_ip_) between the BU2·Cl^−^ complex and TBA^+^ to be 1.2 × 10^5^ M^−1^ (Fig. S27†). Although we do not know the value of *K*_ip_ of TBACl in chloroform, we expect it to be about the same order of magnitude as the published *K*_ip_ value of 9 × 10^9^ M^−1^ for TEACl in the same solvent.^22^ The found *K*_ip_ value of 1.2 × 10^5^ M^−1^ for the BU2·Cl^−^ complex and TBA^+^ is several orders of magnitude lower than that for TEACl, showing that the macrocycle dramatically weakened the ion pairing. Moreover, the ITC experiments performed in acetonitrile and chloroform showed similar preferences of BU1 for Br^−^ over NO_3_^−^. As ion-pairing in acetonitrile is weak (*K*_ip_ for TBACl is 5 M^−1^),^22^ we believe that this experiment further supports the insignificance of cations in the observed preferential anion binding.

**Fig. 3:**
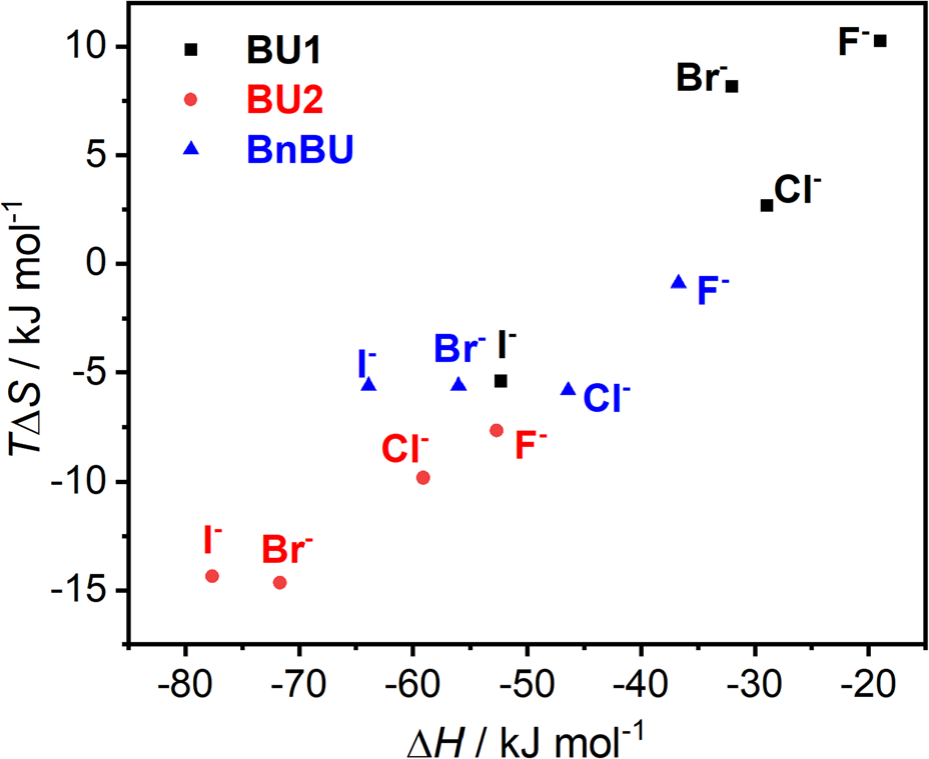
Enthalpy–entropy compensation plot of the BU complexes with halides.

The observed differences in binding affinities between BnBU, BU1, and BU2 are reflected in their enthalpy–entropy compensation (Fig. 3). All complexes are strongly exothermically favorable, but most of them are counterbalanced by an entropic cost. The enthalpy–entropy compensation is most significant for BU2 halide complexes and the least for the complexes of BU1.

**Fig. 4:**
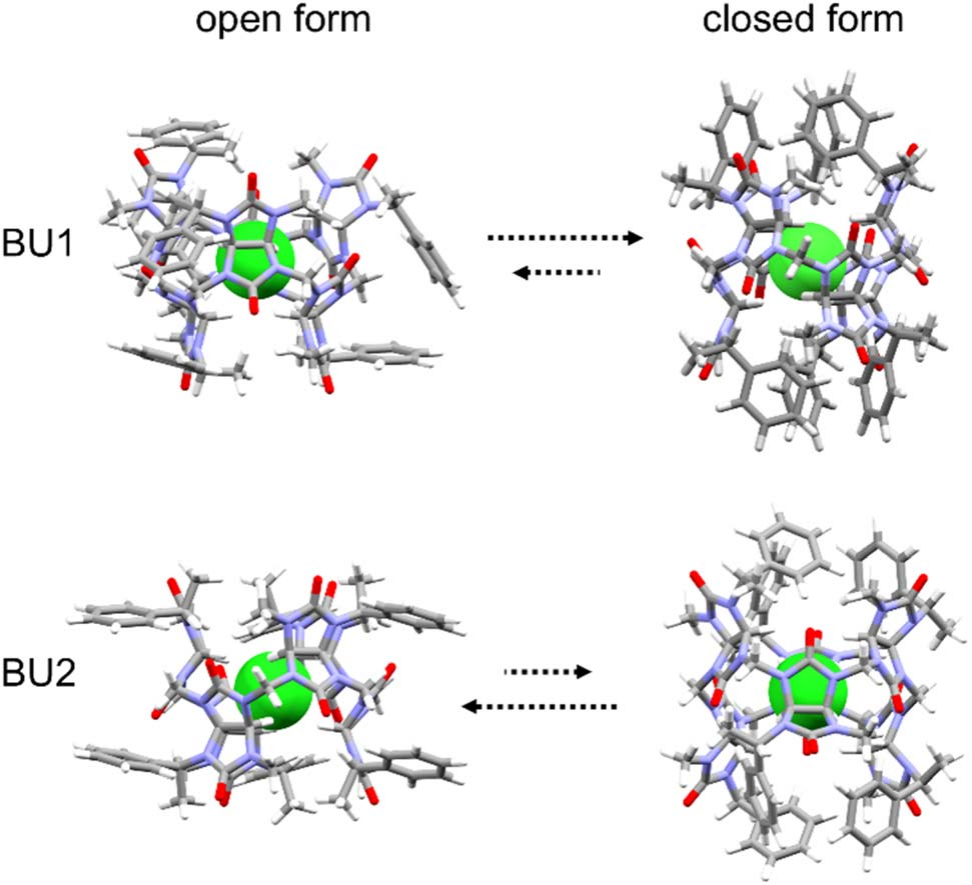
Two boundary conformations for BU1 and BU2.

We performed a computational study to better understand bambusuril binding affinity and selectivity differences. Quantum-chemical calculations were performed for the macrocycles with empty cavities and their complexes with halides, all in implicit chloroform solvent. Bambusurils BU1 and BU2 are diastereomers to each other. They differ only in the placement of the 1-phenylethyl and methyl groups, which are attached to the nitrogen atoms of the glycoluril building blocks in opposite positions (see *R*^1^ and *R*^2^ in Fig. 1). In addition to these distinct arrangements, the 1-phenylethyl groups can rotate along the N–C bond, resulting in different conformers of the macrocycles. Here, we tested two boundary conformations (Fig. 4), which differ by the orientation of the methyl and phenyl groups of the 1-phenylethyl substituent relative to glycoluril five-membered rings.

In the closed form, the aromatic parts of 1-phenylethyl groups are aligned with the axial axis of the macrocycle, seemingly “closing” the cavity.

However, a short MD simulation reveals that the cavity is not effectively sealed but rather extended due to the tumbling of the phenyl groups. The cavity is closed upon halide binding. In contrast, the aromatic parts head out of the cavity in the open form, exposing the cavity to the solvent, similar to non-derivatized bambusurils.^23^ In all cases, the calculations showed that BU1 prefers the closed form, while BU2 prefers the open form (Fig. 4). The magnitude of the preference is comparable to the binding affinities, and it does not depend on the type of anion occupying their cavity (Table S5†). The calculation agrees with the previously determined crystal structure of the BU2 complex with Br^−^,^20^ in which the macrocycle adapts the open-form conformation. The stable conformations, the closed for BU1 and the open for BU2, while completely different, share the same structural feature. In both cases, the methine group of the 1-phenylethyl group interacts with carbonyl oxygens at the central belt through hydrogen bonding. This seems to be a key interaction already observed in the other BU derivatives, such as BnBU or dodecamethylbambusuril.^19,20^

Next, we calculated the binding affinities of halide anions to BU1 and BU2. In agreement with the experiment, we found that BU2 exhibits binding affinities higher than BU1 (Table 2). Compared to experimental values, the calculated values are overestimated because they do not include entropy contributions. Better agreement can be observed for the calculated (ΔΔ*E*_b_) and experimental (ΔΔ*G*_b_) binding energy differences between BU2 and BU1. In addition, to trace the origin of this preference, we decomposed the obtained binding affinities (ΔΔ*E*_b_) into (i) the interaction of the halide anion with the bambusuril cavity (ΔΔ*E*_i_), (ii) macrocycle deformation upon halide anion binding (ΔΔ*E*_d_), and (iii) solvation contributions (ΔΔ*E*_s_). Their values clearly reveal that the binding difference between BU1 and BU2 is predominantly the result of interaction factors, as solvation and deformation effects do not substantially differ for different anions.

**Table 2:** Experimental (Δ*G*_b_) and calculated (Δ*E*_b_) binding affinities for BU1 and BU2 halide complexes. The binding affinity difference (ΔΔ*G*_b_ and ΔΔ*E*_b_) between BU2 and BU1 and its decomposition into the interaction ΔΔ*E*_i_, the deformation ΔΔ*E*_d_, and the solvation ΔΔ*E*_s_ contributions. All energies are in kJ mol^−1^

Anion	BU1	BU2	BU2–BU1	BU1	BU2	BU2–BU1			
	Δ*G*_b_	Δ*G*_b_	ΔΔ*G*_b_	Δ*E*_b_	Δ*E*_b_	ΔΔ*E*_b_	ΔΔ*E*_i_	ΔΔ*E*_d_	ΔΔ*E*_s_
F^−^	−29.7	−43.5	−13.7	−45.4	−65.1	−19.7	−31.4	8.0	3.7
Cl^−^	−31.6	−48.5	−16.8	−85.1	−103.9	−18.8	−26.3	1.6	5.9
Br^−^	−41.4	−51.6	−10.2	−95.7	−106.3	−10.5	−19.6	3.0	6.0
I^−^	−47.5	−61.9	−14.4	−108.0	−113.7	−5.7	−10.5	3.4	1.5

We tried to decipher possible structural factors responsible for the observed binding preference. The differences in the preferred conformation of BU1 and BU2 are clearly caused by the placement of 1-phenylethyl groups and impact the cavity arrangement. The BU1 cavity is screw-like distorted, as indicated by the different lengths of C–H⋯X^−^, by about 0.3 Å for each of the two methine protons of glycoluril units (Table S6†). In contrast, all C–H⋯X^−^ hydrogen bonds in BU2 are equalized with the same length. A similar situation can be found in the experimental structure of BnBU complexed with chloride,^19^ where all C–H⋯Cl^−^ hydrogen bonds are equalized with the same length of 3.13 Å. This result confirms that the structure of the BU2 macrocycle is more flexible compared to BU1 and better adapts to the anion size. On the other hand, the spacious arrangement of the 1-phenylethyl units in BU1 induces distortion of the macrocycle cavity, resulting in a significantly lower anion binding affinity of this macrocycle compared to BU2 and BnBU.^22–31^

## Conclusions

In conclusion, we showed that the position of methyl and 1-phenylethyl groups on the glycoluril building blocks of diastereomeric bambusurils BU1 and BU2 induces significant differences in their anion binding. BU1 binds inorganic anions up to three orders of magnitude less strongly than BU2. Quantum-chemical calculations revealed that these binding differences are due to distortion of the BU1 cavity, which is forced by the arrangement of spacious 1-phenylethyl groups, while the more relaxed arrangement is proposed for the BU2 diastereomer. Moreover, both BU1 and BU2 and previously reported BnBU feature very different or even reverse selectivity for pairs of inorganic anions. This is a unique achievement in bambusuril chemistry, as, until today, different substituents on the macrocycle portals resulted only in the modulation of their binding affinity, while selectivity among anions remained similar. Thus, our results show that bulky substituents could be used to rigidify the structures of any flexible macrocyclic receptor to tune its binding selectivity toward guest molecules.

## Data availability

The data supporting this article have been included as part of the ESI.†

## Author contributions

V. S. conceived the project. J. S. synthesized the macrocyclic compounds. C. R. and S. G. conducted ITC measurements. S. G. performed NMR experiments. P.K. carried out the computational studies. V. S. and P. K. drafted the manuscript. All authors contributed to the writing of the ESI† and to the revision of the manuscript.

## Conflicts of interest

There are no conflicts to declare.

## Supplementary Material

SC-OLF-D4SC07150F-s001
