# Supplementary material for: Reversing selectivity of bambusuril macrocycles toward inorganic anions by installing spacious substituents on their portals
Source: Chem Sci. 2024 Dec 6;16(3):1288–92. doi: 10.1039/d4sc07150f (PMC11639902; doi:10.1039/d4sc07150f)
Supplement: SC-016-D4SC07150F-s001 [file SC-016-D4SC07150F-s001.zip › corr-corr-25-11-esi.pdf]

Electronic Supplementary Information

Reversing Selectivity of Bambusuril Macrocycles toward Inorganic Anions by Instaling Spacious Substituents on Their Portals

Carola Rando, Surbhi Grewal, Jan Sokolov, Petr Kulhánek, Vladimír Šindelář

Table of Contents

General Information……………………………………………………………………………………………………..S3

Isothermal Titration Calorimetry (ITC)……………………………………………………………………………...S3

NMR..S28

DOSY... S29

Dilution experiment... S30

Computational Methods...S31

General information

All reagents and solvents were purchased from commercial suppliers and used without further purification. For all investigated anions, tetrabutylammonium (TBA) salts were used for the supramolecular studies. Macrocycles BU1, BU2, and BnBU were prepared according to previously reported protocols.[1,2]

ITC analysis was recorded on MicroCal VP-ITC from Malvern.

Isothermal Titration Calorimetry (ITC)

A study employing isothermal titration calorimetry (ITC) was carried out using a MicroCal VP-ITC instrument from Malvern. The experiments were conducted at 298.15 K in pure chloroform or acetonitrile. The heat responses recorded during the titration process are depicted in the upper graph of each figure within this section. Each peak on the graph corresponds to the introduction of a 10 μL salt solution into the cell containing chiral BU alone or complexed with the competitor. The number of injections for each measurements was 29. In general, the lower graph illustrates the cumulative heat released as a function of the total concentration of the ligand. The solid line on the graph corresponds to the best-fit line obtained through a least-squares analysis of the data. To analyse the integrated heat effects, a single-site model was employed in nonlinear regression analysis. The association constant K_a and the standard binding enthalpy $\Delta H°$ were achieved using experimental data matched to a theoretical titration curve. The standard free energy $\Delta G°$ and standard entropy $\Delta S°$ were obtained through the equation: $\Delta G° = \Delta H° - T\Delta S° = -RT \ln K_a$, where T is the absolute temperature and R is the molar gas constant (8.3145 J mol^{-1} K^{-1}).

Competition experiments were performed to measure the association constant of a tightly bound complexes. The stronger guest (from the syringe) was injected into the complex solution formed by the BU and the weaker guest. We employed the model supplied with the instrument that takes into consideration the change of concentration of all reagents. Acetate, methanesulfonate, and chloride salts were used as a guest for the competition. The use of two step determination of association constants by competition experiment increases the uncertainty of obtained absolute values, but it mitigates the influence of dilution of competing guest which has to be present in higher concentration in the measuring cell. This effect is significant in solvents where the dissociation of tight ion pairs is limited. The effect of using multiple competing anions was evaluated by cross experiments that proved the validity of this approach.

ITC measurements

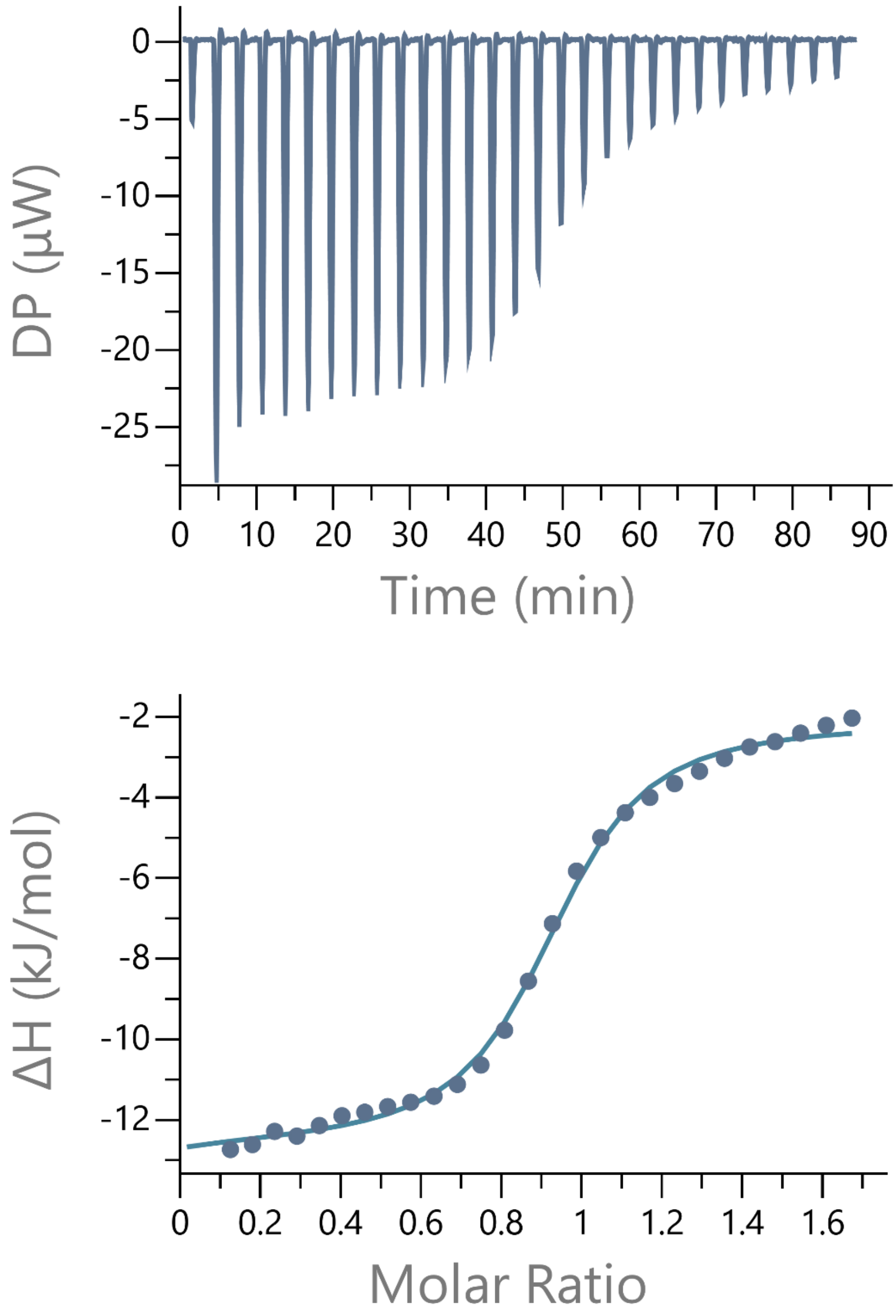

Figure S1. ITC titration of BU2 (0.68 mM) with tetrabutylammonium (TBA) fluoride (5.21 mM) in the presence of $TBAMeSO_3$ (1 mM) in chloroform.

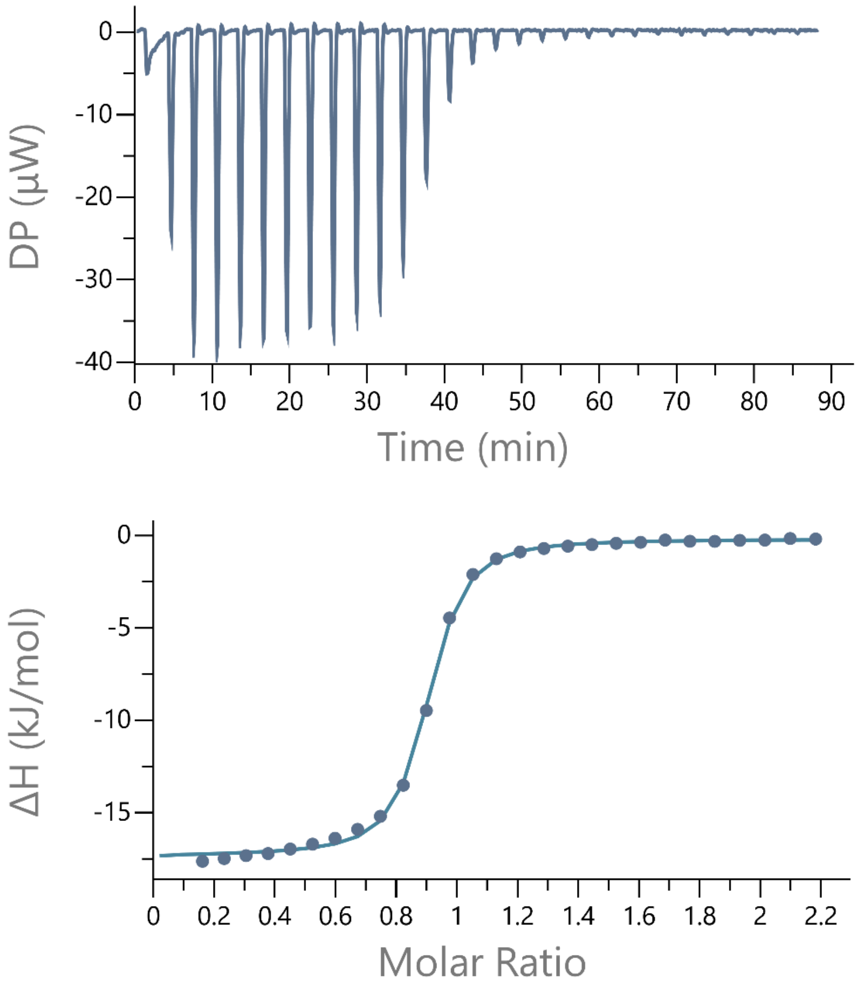

Figure S2. ITC titration of BU2 (0.5 mM) with tetrabutylammonium (TBA) chloride (5 mM) in the presence of $TBAMeSO_3$ (1 mM) in chloroform.

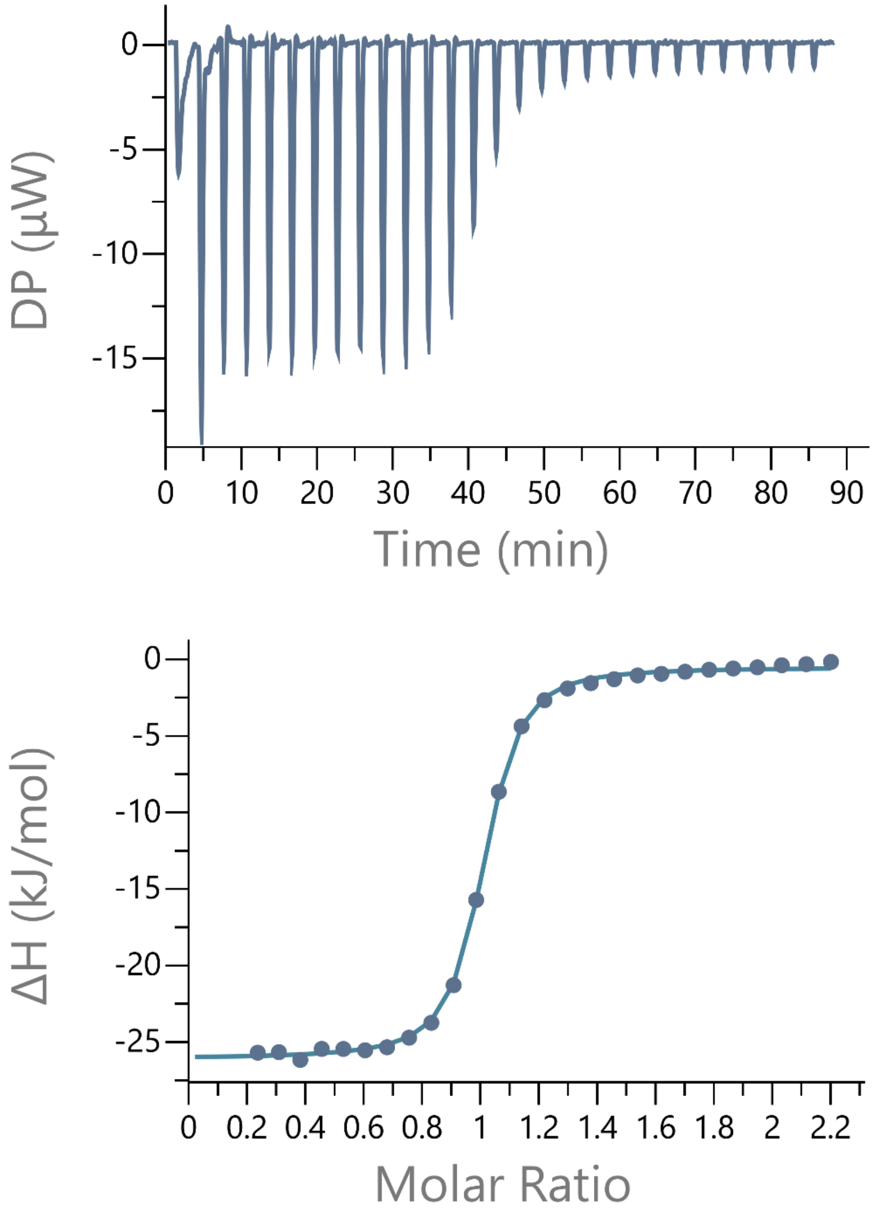

Figure S3. ITC titration of BU2 (0.126 mM) with tetrabutylammonium (TBA) acetate (1.27 mM) in chloroform.

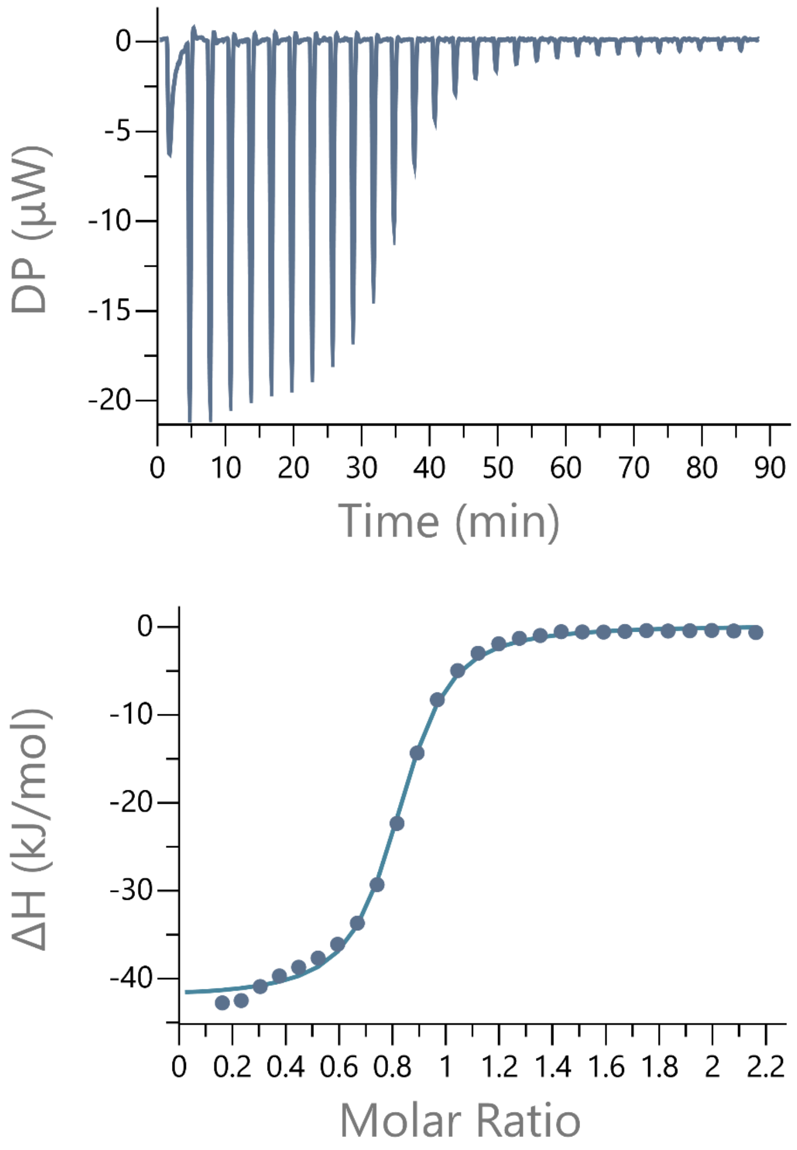

Figure S4. ITC titration of BU2 (0.1 mM) with tetrabutylammonium (TBA) methanesulfonate (0.99 mM) in chloroform.

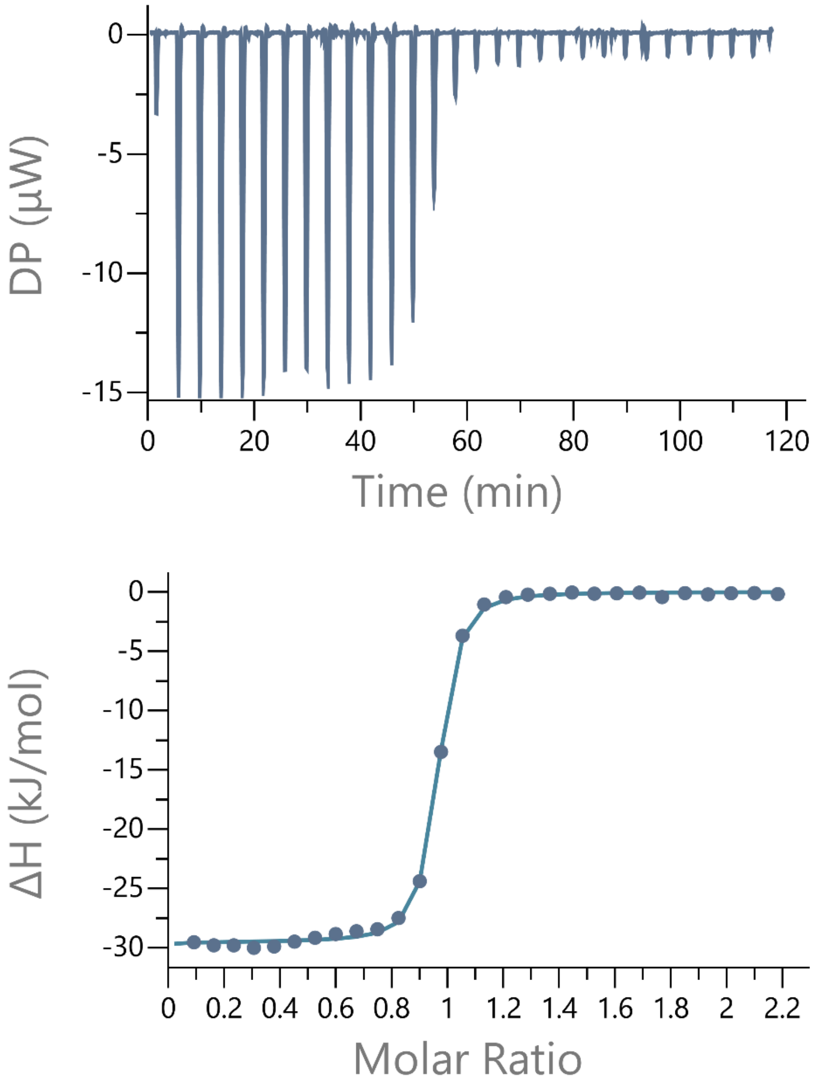

Figure S5. ITC titration of BU2 (0.1 mM) with tetrabutylammonium (TBA) nitrate (1 mM) in the presence of TBAacetate (0.2 mM) in chloroform.

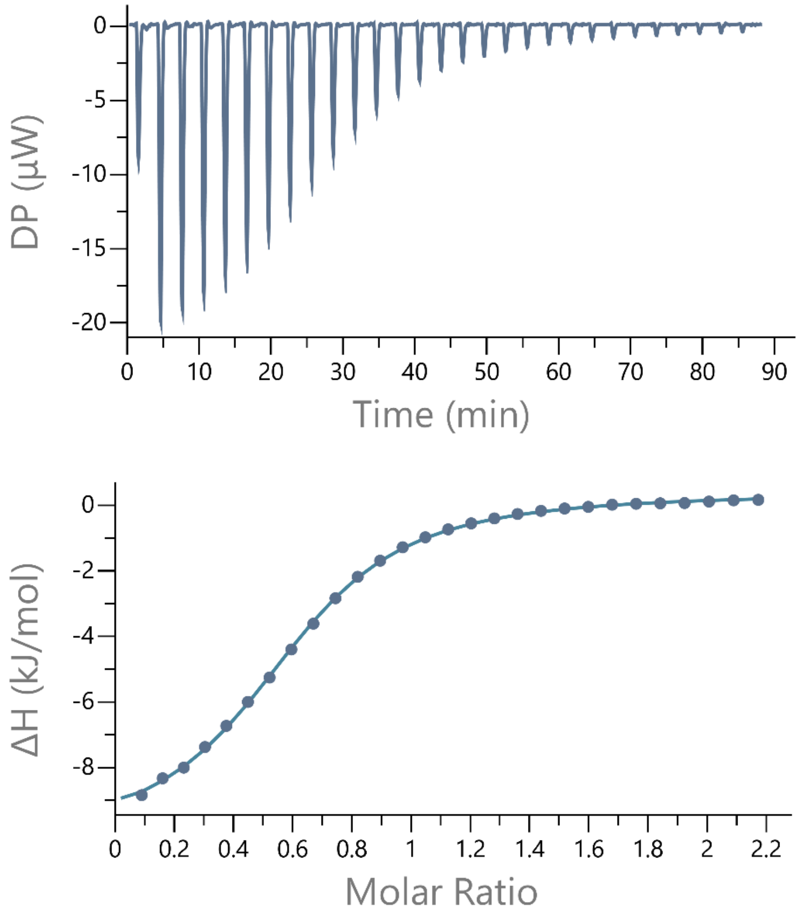

Figure S6. ITC titration of BU2 (0.5 mM) with tetrabutylammonium (TBA) perrhenate (4.98 mM) in the presence of TBAMeSO$_3$ (1 mM) in chloroform.

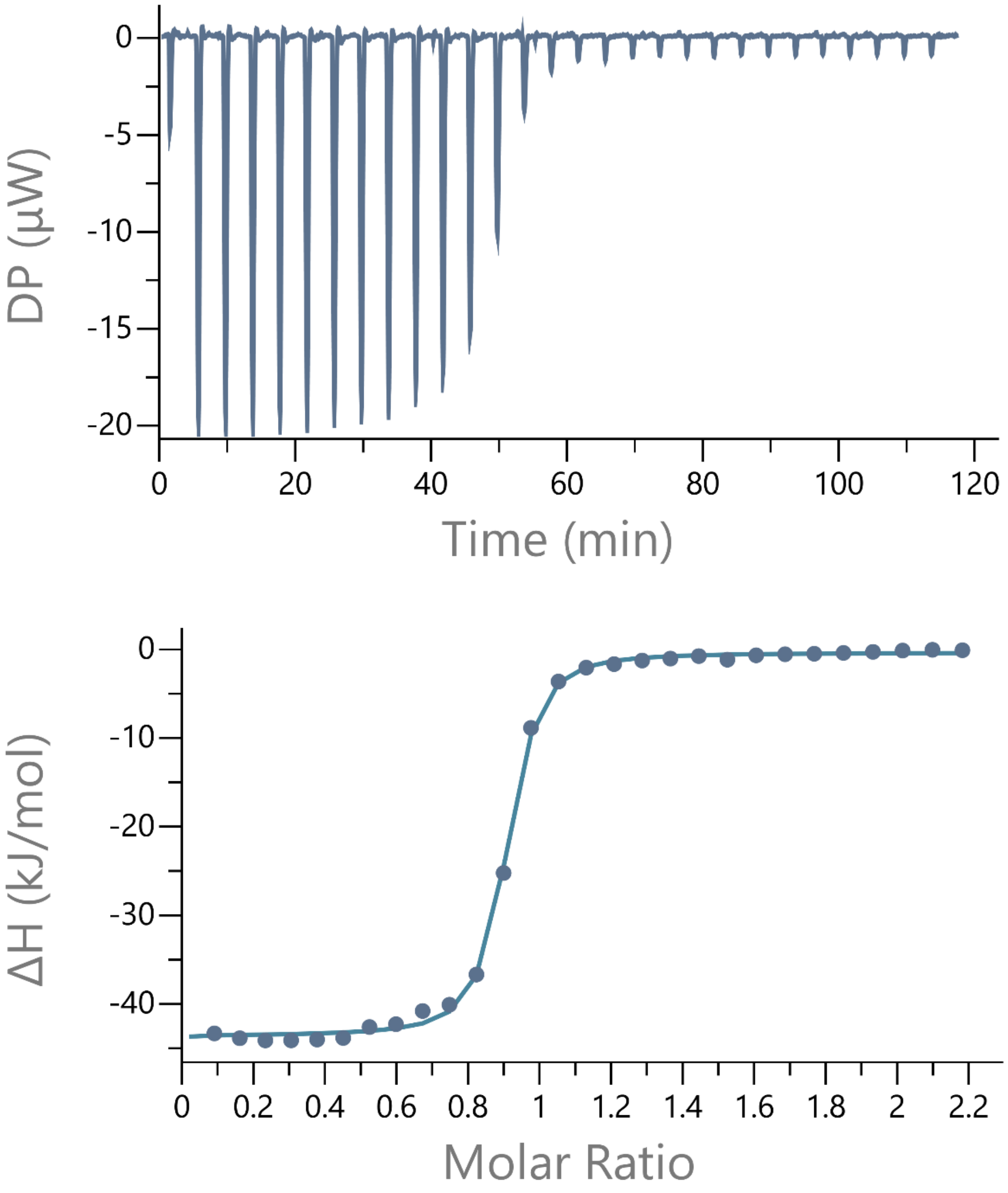

Figure S7. ITC titration of BU2 (0.1 mM) with tetrabutylammonium (TBA) perchlorate (1 mM) in the presence of TBAacetate (0.2 mM) in chloroform.

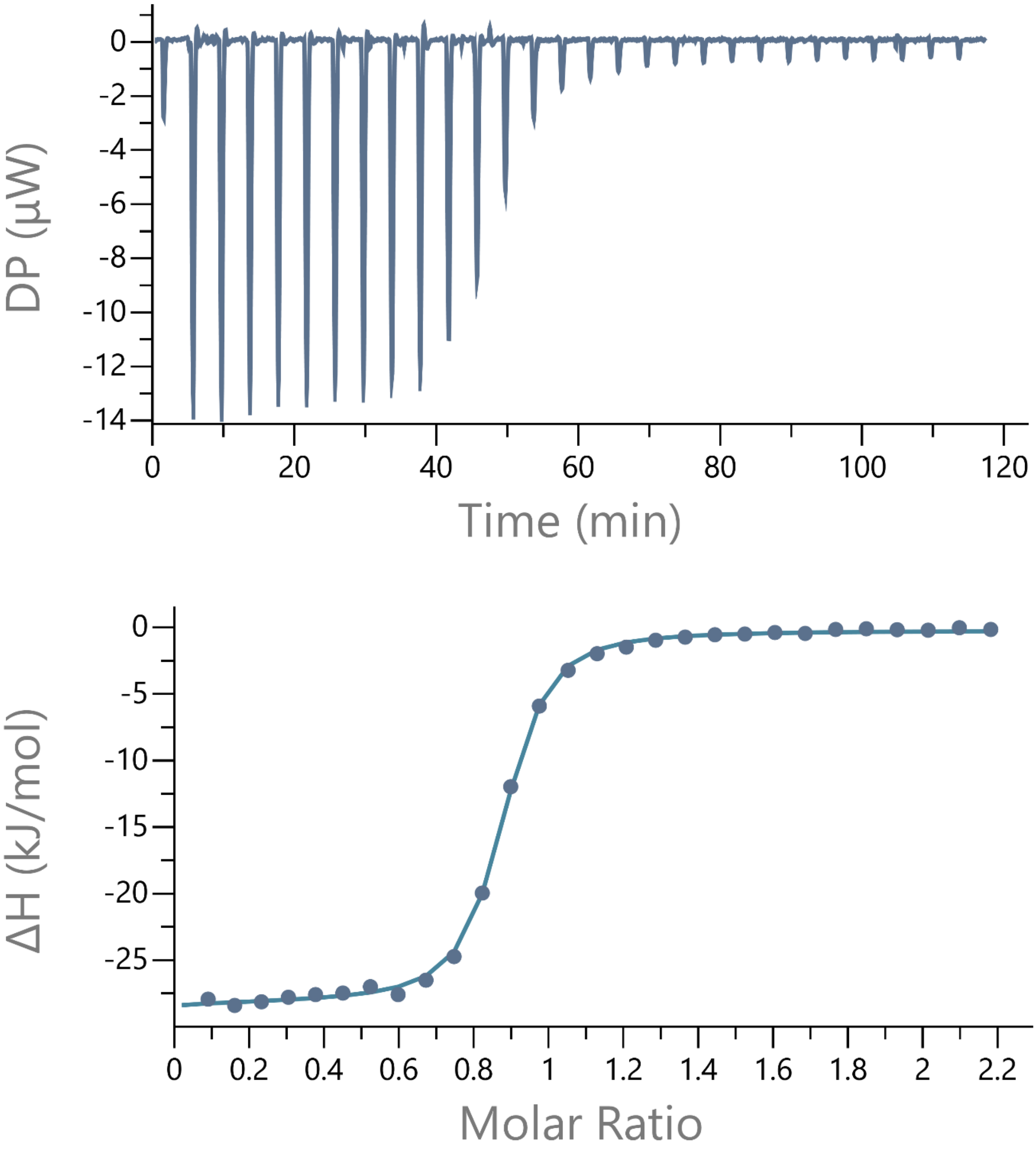

Figure S8. ITC titration of BU2 (0.1 mM) with tetrabutylammonium (TBA) hexafluorophosphate (1 mM) in the presence of TBAMeSO$_3$ (0.2 mM) in chloroform.

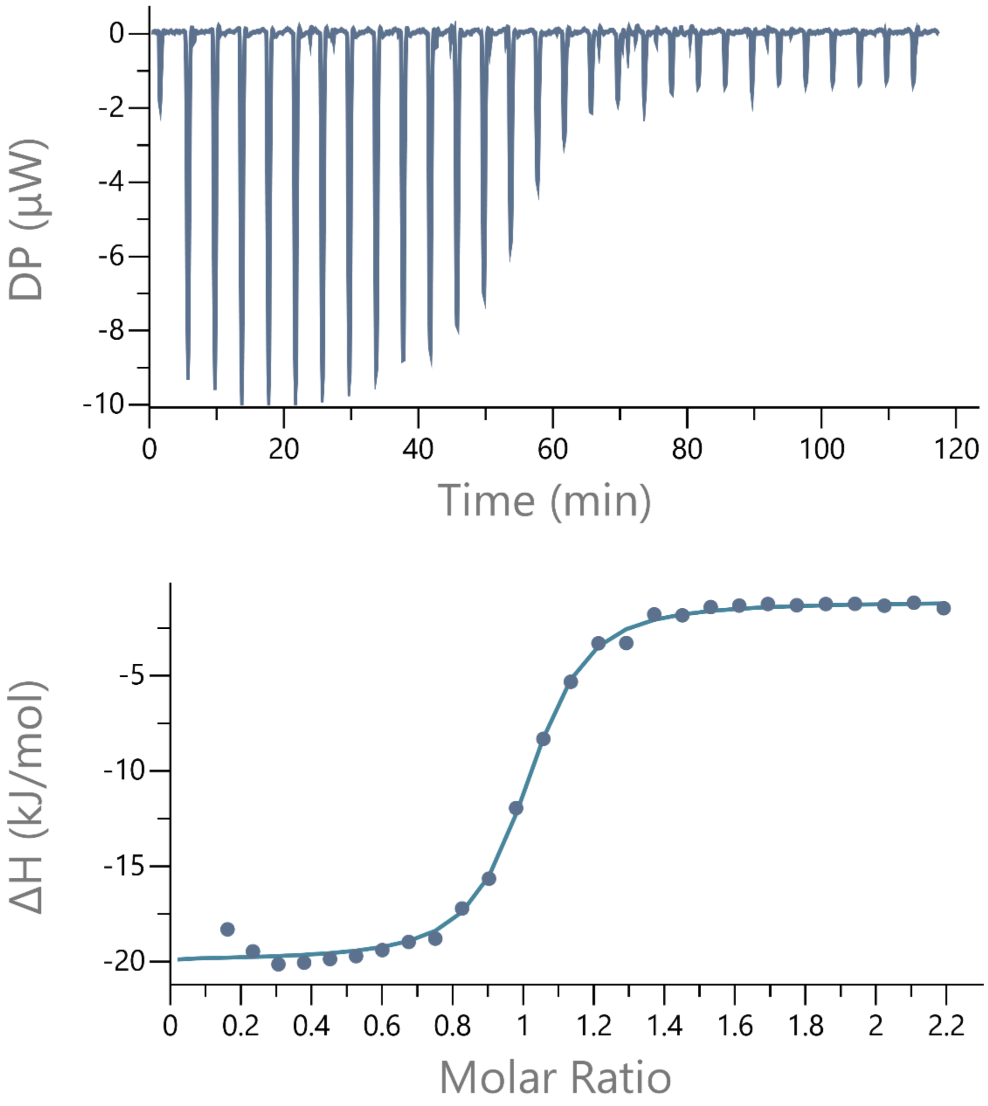

Figure S9. Competitive ITC titration of BU2 (0.1 mM) with tetrabutylammonium (TBA) iodide (1 mM) in the presence of TBACl (0.2 mM) in chloroform.

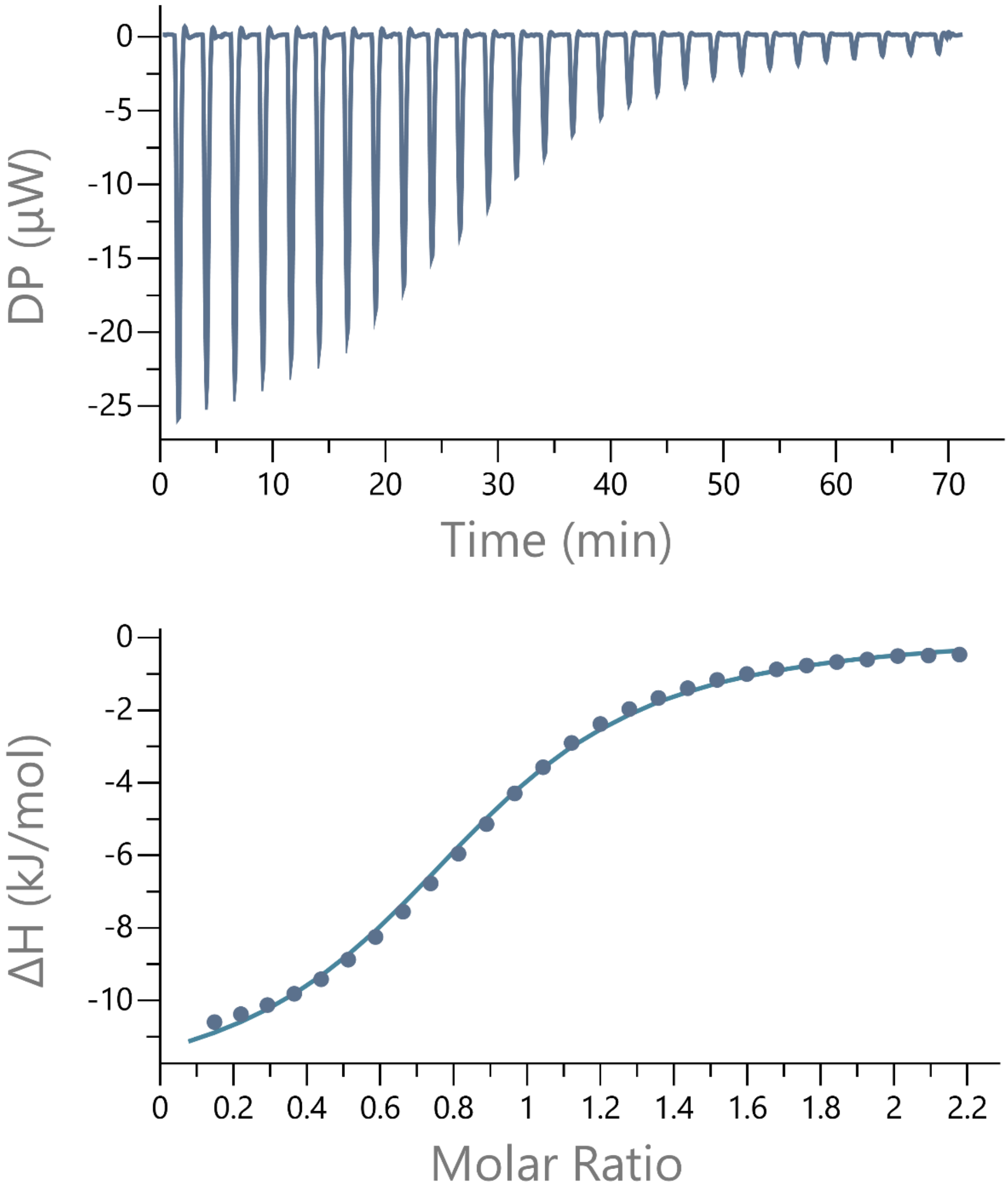

Figure S10. Competitive ITC titration of BU2 (0.5 mM) with tetrabutylammonium (TBA) bromide (5.03 mM) in the presence of TBACl (1.3 mM) in chloroform.

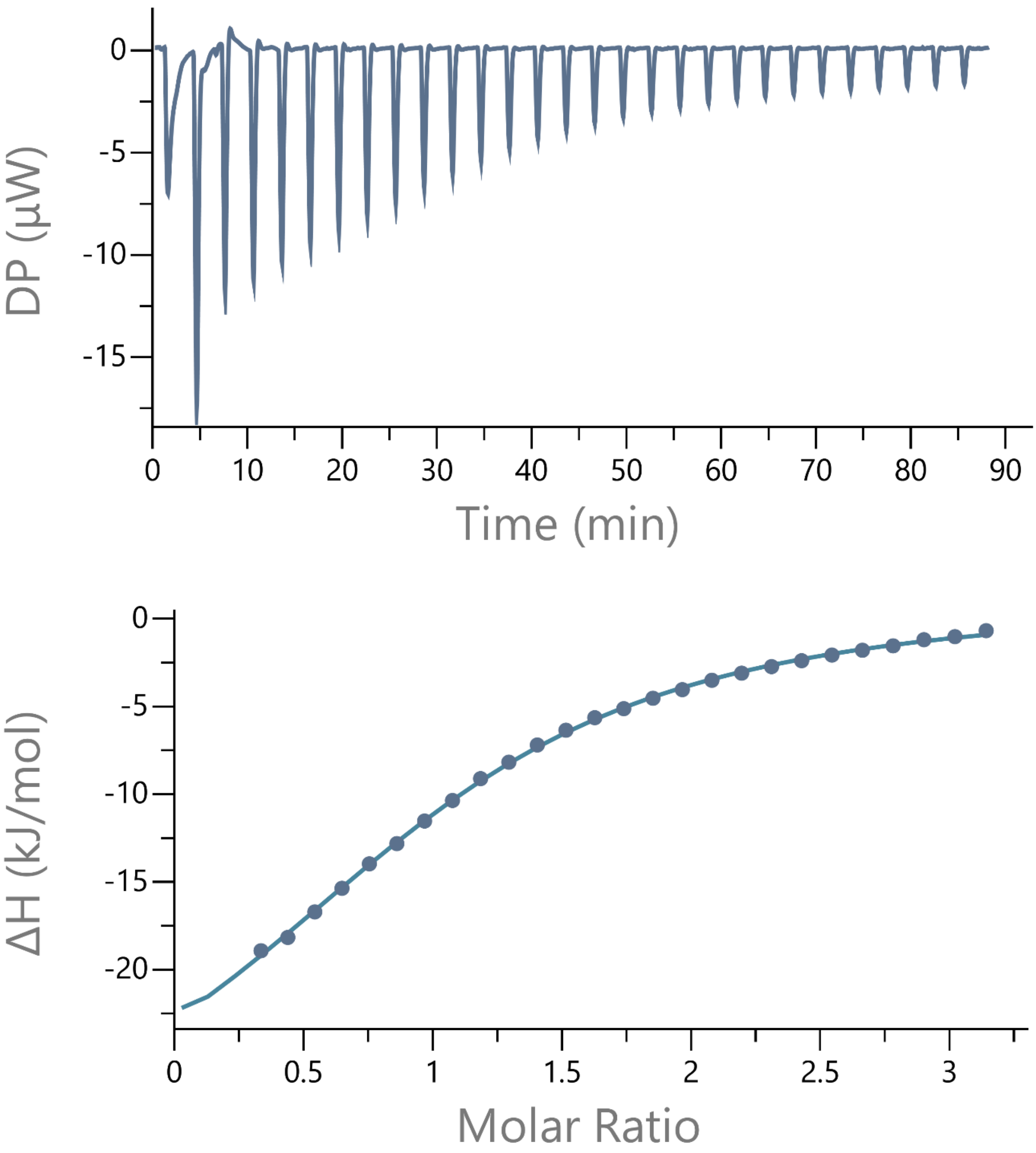

Figure S11. ITC titration of BU1 (0.1 mM) with tetrabutylammonium (TBA) acetate (1.44 mM) in chloroform.

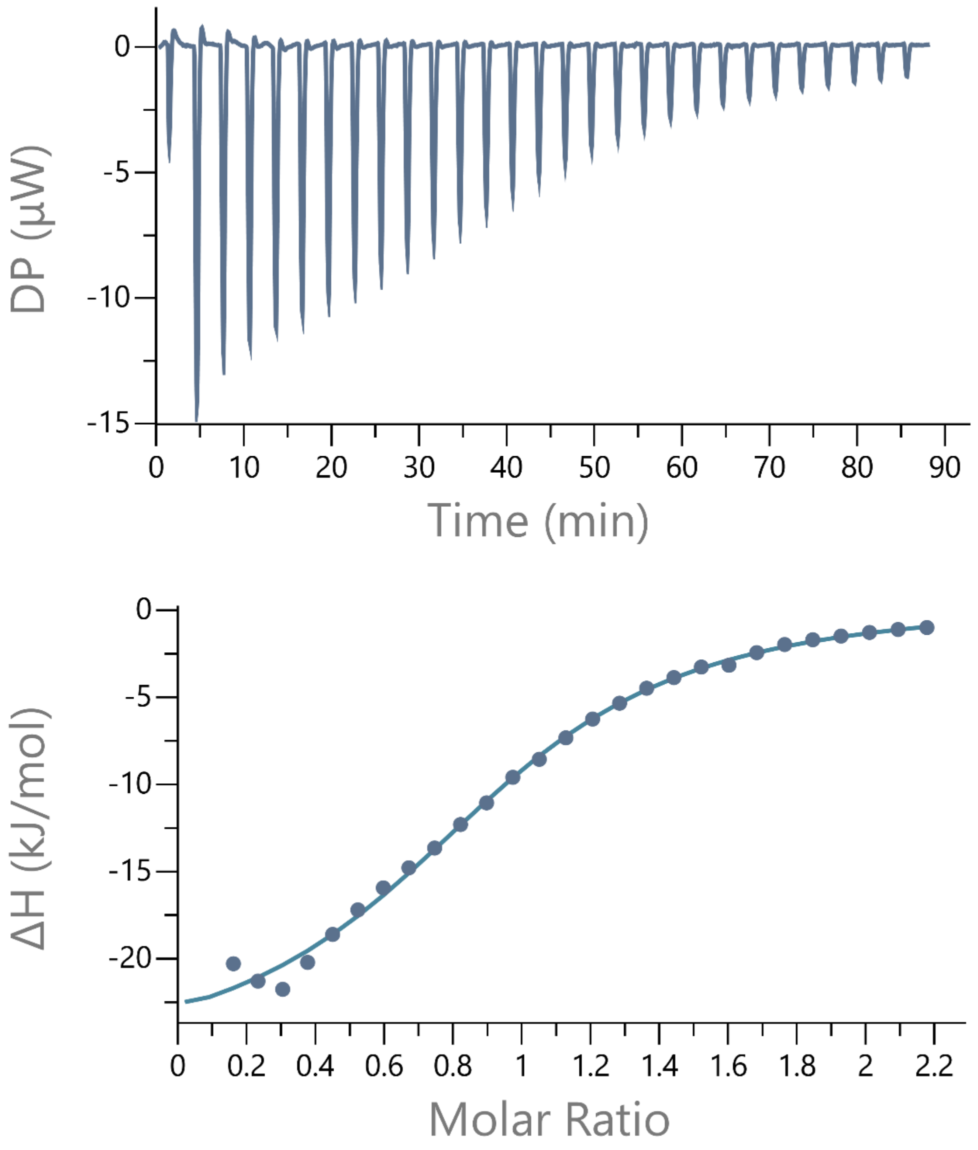

Figure S12. ITC titration of BU1 (0.1 mM) with tetrabutylammonium (TBA) methanesulfonate (0.998 mM) in chloroform.

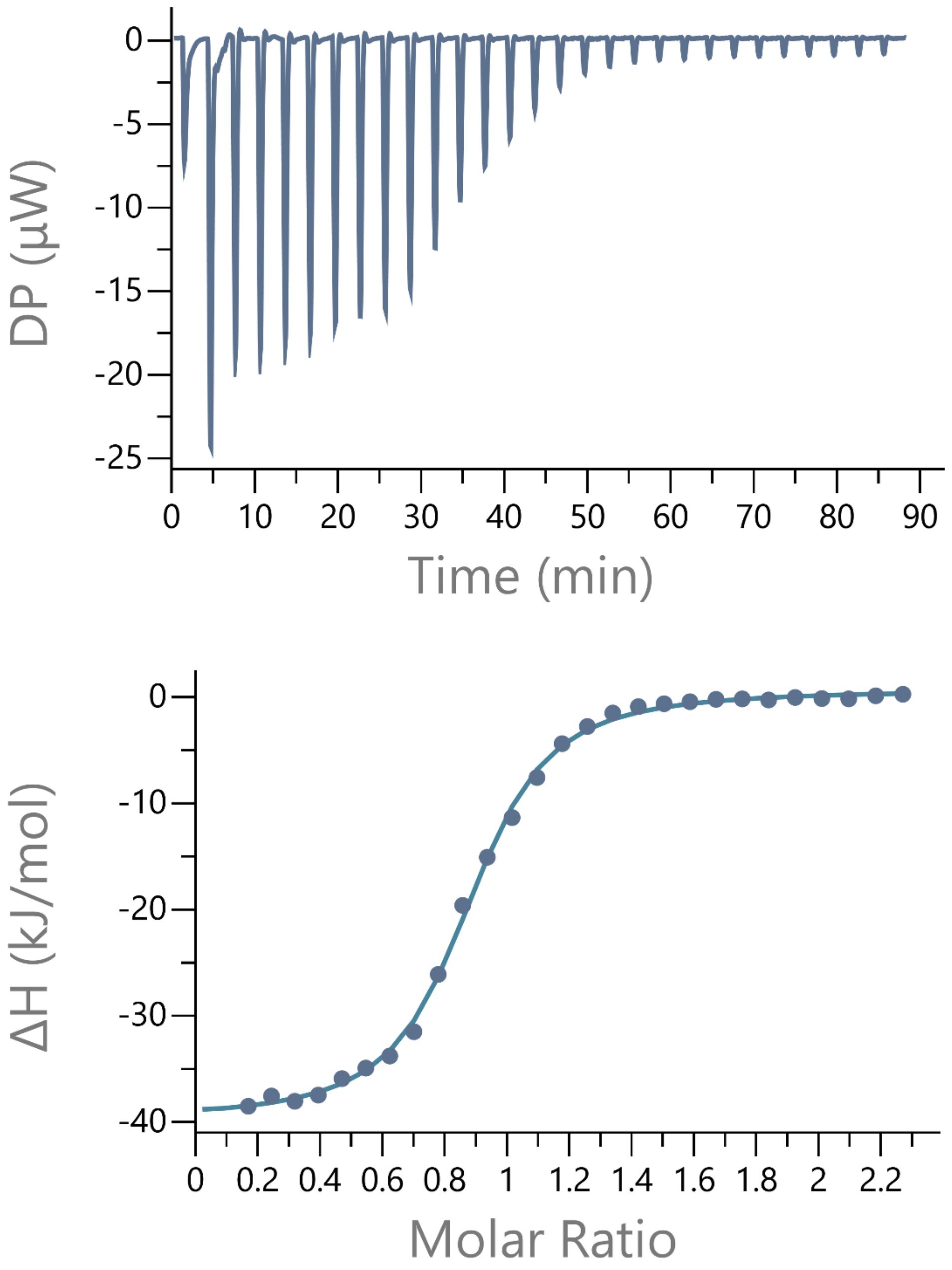

Figure S13. ITC titration of BU1 (0.1 mM) with tetrabutylammonium (TBA) nitrate (1.04 mM) in chloroform.

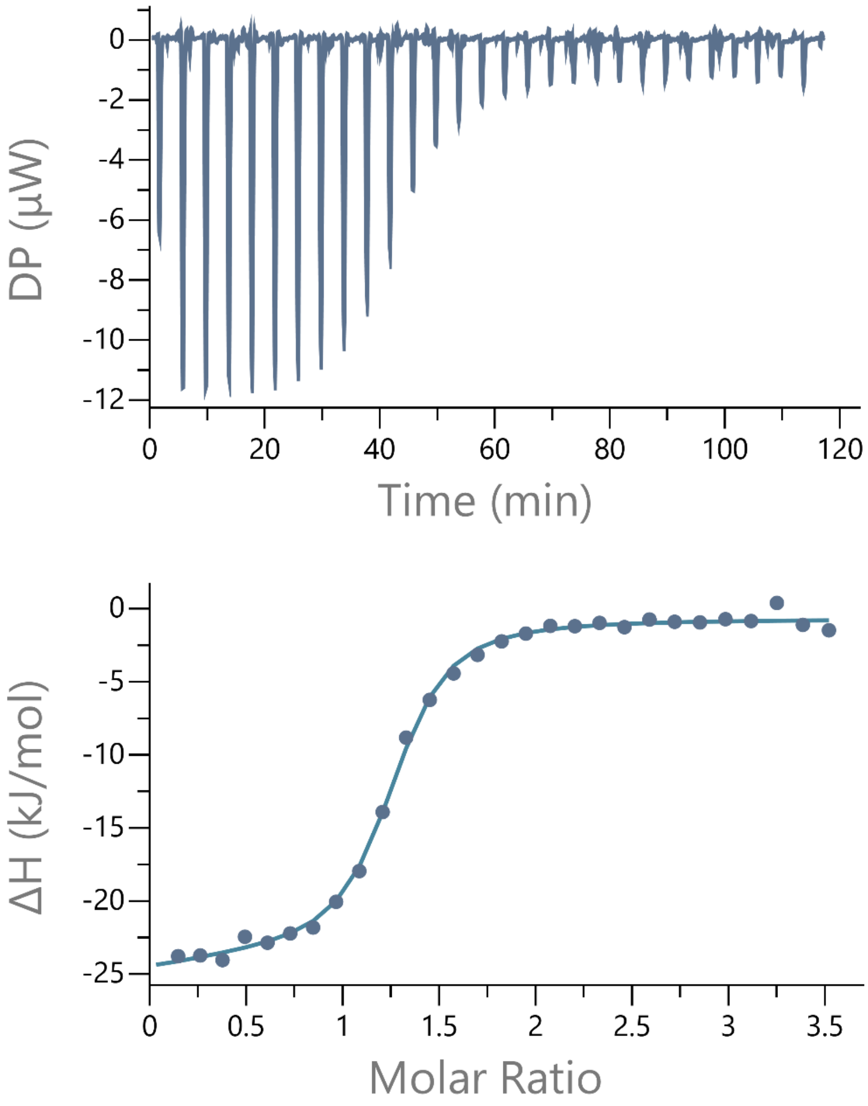

Figure S14. ITC titration of BU1 (0.062 mM) with tetrabutylammonium (TBA) perrhenate (1 mM) in the presence of $TBAMeSO_3$ (0.2 mM) in chloroform.

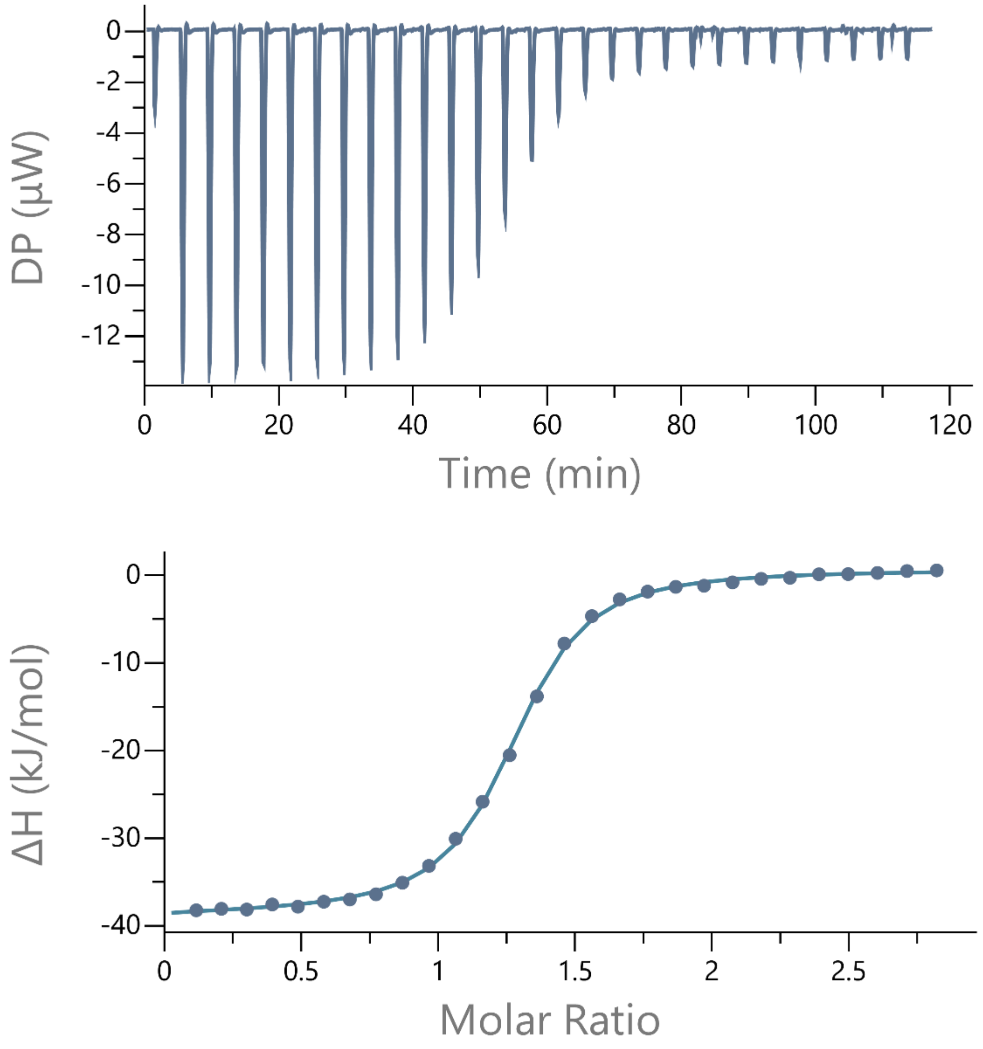

Figure S15. ITC titration of BU1 (0.058 mM) with tetrabutylammonium (TBA) perchlorate (0.750 mM) in the presence of TBACl (0.2 mM) in chloroform.

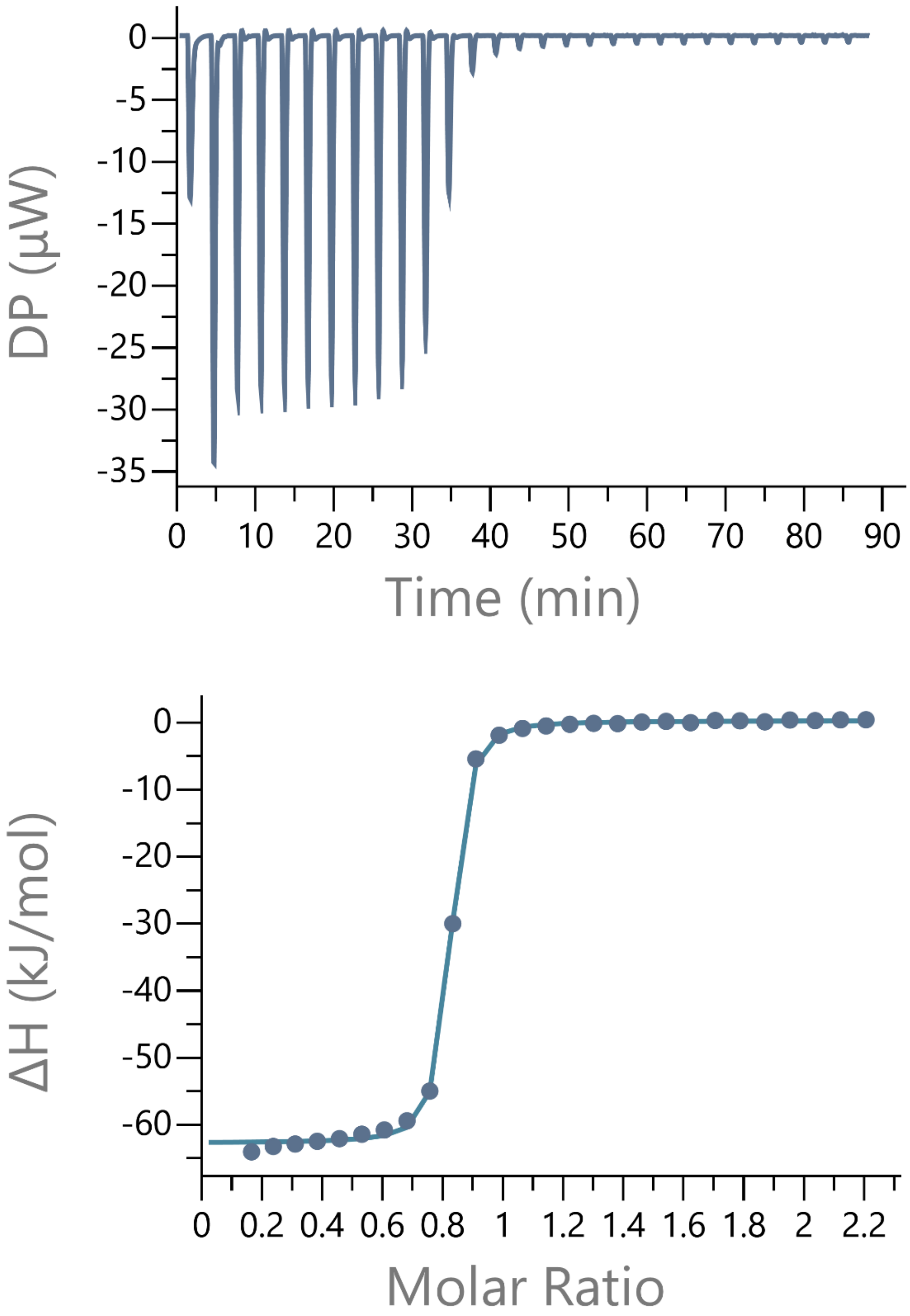

Figure S16. ITC titration of BU1 (0.1 mM) with tetrabutylammonium (TBA) hexafluorophosphate (1.01 mM) in chloroform.

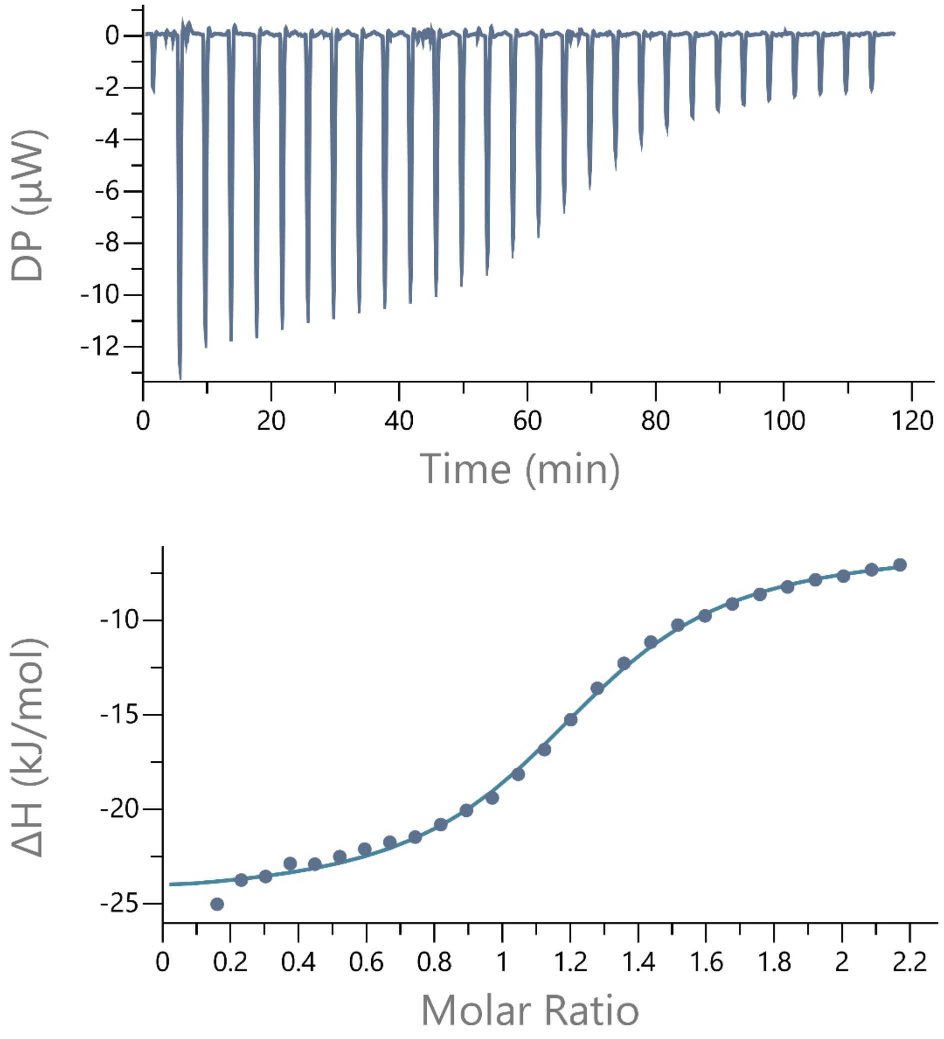

Figure S17. ITC titration of BU1 (0.142 mM) with tetrabutylammonium (TBA) fluoride (1.41 mM) in chloroform.

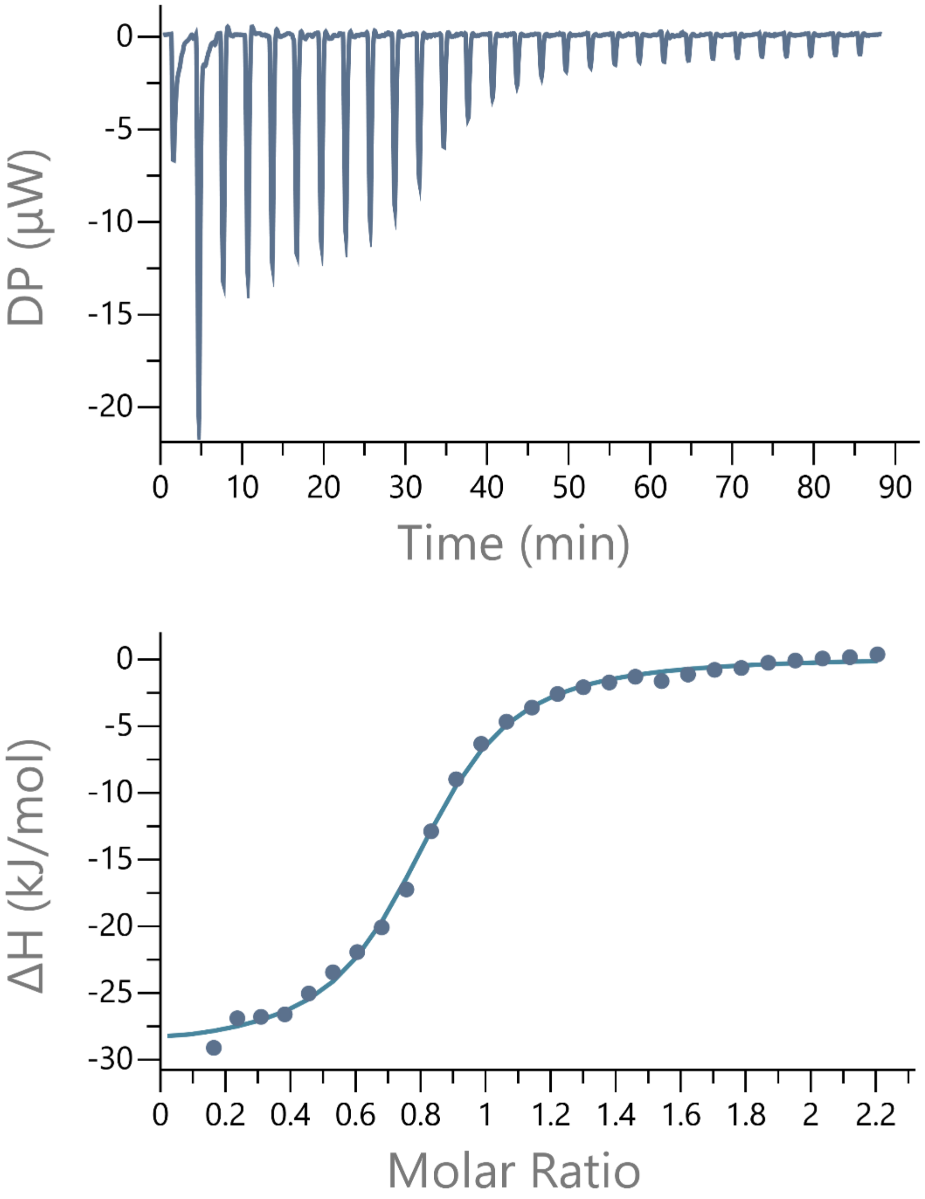

Figure S18. ITC titration of BU1 (0.1 mM) with tetrabutylammonium (TBA) chloride (1.01 mM) in chloroform.

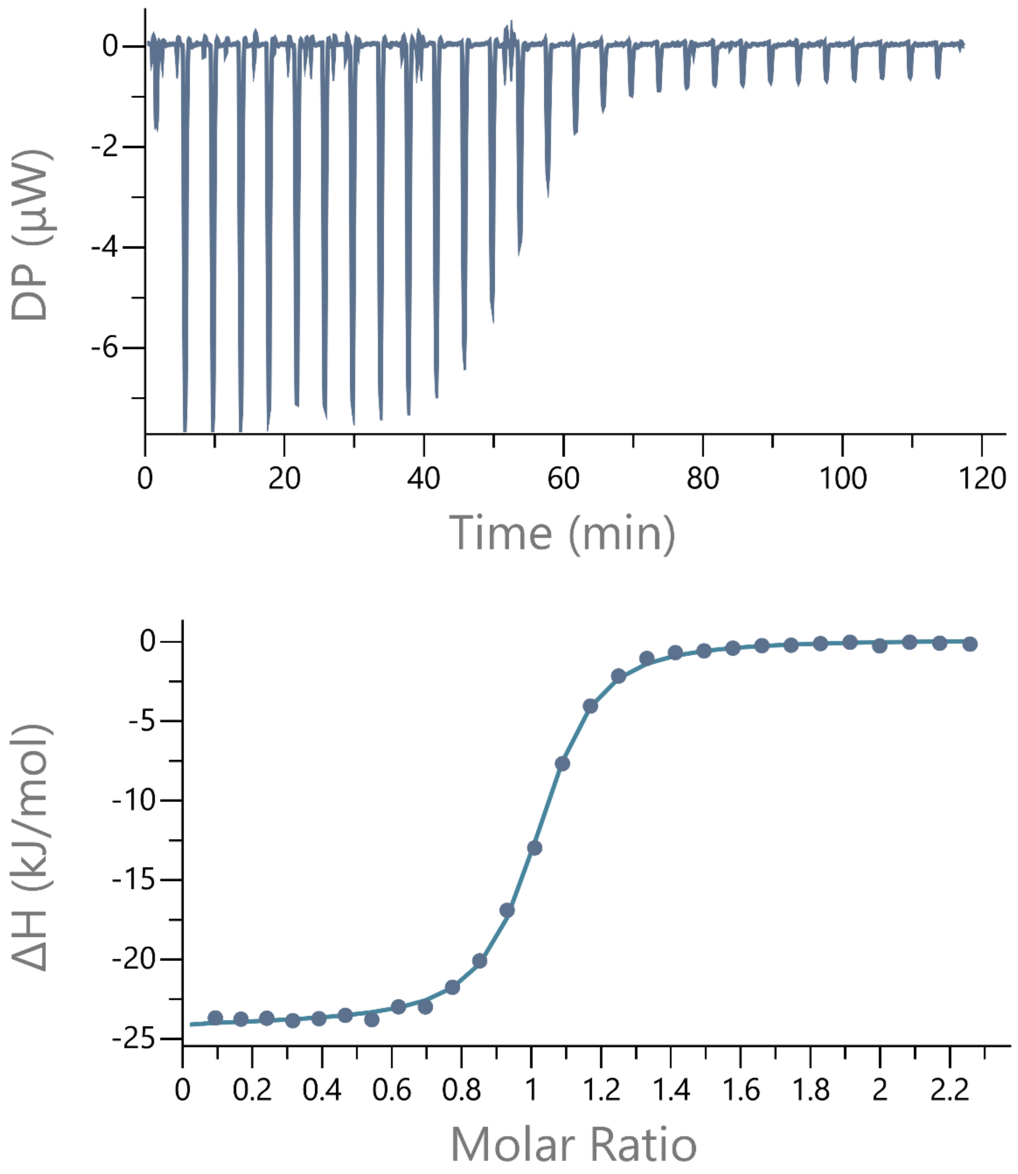

Figure S19. ITC titration of BU1 (0.058 mM) with tetrabutylammonium (TBA) iodide (0.6 mM) in the presence of TBACl (0.2 mM) in chloroform.

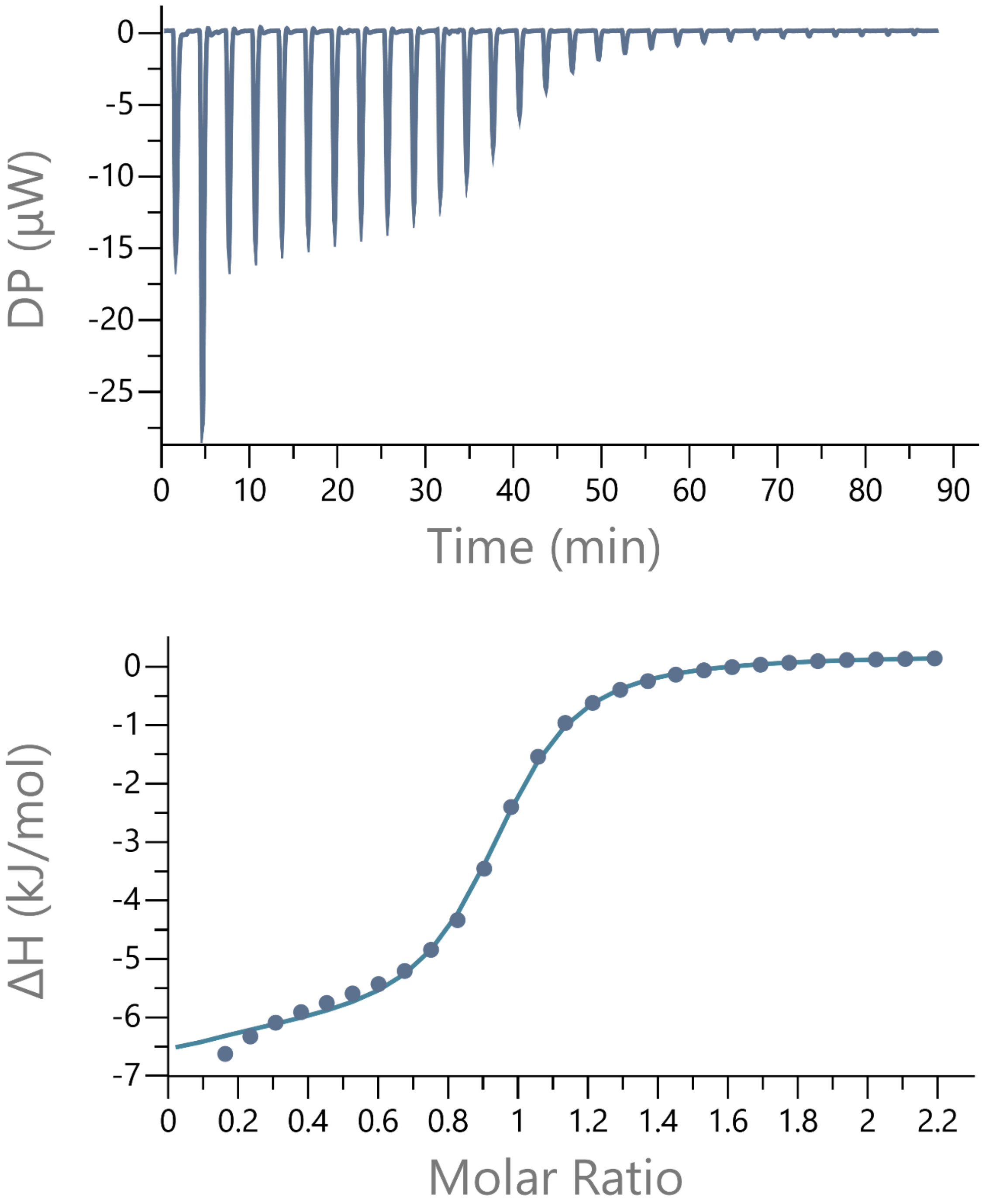

Figure S20. ITC titration of BU1 (0.5 mM) with tetrabutylammonium (TBA) bromide (5.02 mM) in the presence of $TBAMeSO_3$ (1 mM) in chloroform.

Table S1 Apparent association constants (K_a), binding stochiometry (N), and thermodynamical parameters (ΔH, $T\Delta S$, ΔG) of the tetrabutylammonium (TBA) anion⊂BU1 complexes determined by ITC in chloroform at 298.15 K

Anion	N	ΔH (kJ mol^{-1})	$T\Delta S$ (kJ mol^{-1})	K_a (M^{-1})	ΔG (kJ mol^{-1})
$CH_3CO_2^-$	1.0	−34.6	−10.1	(1.9±0.3) × 10^4	−24.5
$MeSO_3^-$	0.9	−25.8	+2.3	(8.2±1.2) × 10^4	−28.1
NO_3^-	0.9	−40.6	−8.0	(5.1±0.1) × 10^5	−32.6
ReO_4^-	0.9	−44.7	−3.4	(1.7±0.2) × 10^7	−41.3
ClO_4^-	1.2	−66.9	−22.1	(6.8±0.7) × 10^7	−44.8
PF_6^-	0.7	−63.1	−22.7	(1.2±0.2) × 10^7	−40.4
F^-	1.3	−19.0	+10.2	(1.3±0.2) × 10^5	−29.2
Cl^-	0.8	−28.9	+2.7	(3.3±0.3) × 10^5	−31.6
Br^-	1.0	−32.0	+8.2	(1.1±0.1) × 10^7	−40.2
I^-	1.0	−52.3	−5.4	(1.6±0.1) × 10^8	−46.9

Table S2 Apparent association constants (K_a) , binding stochiometry (N), and thermodynamical parameters (ΔH, $T\Delta S$, ΔG) of the tetrabutylammonium (TBA) anion⊂BU2 complexes determined by ITC in chloroform at 298.15 K

Anion	N	ΔH (kJ mol^{-1})	$T\Delta S$ (kJ mol^{-1})	K_a (M^{-1})	ΔG (kJ mol^{-1})
$CH_3CO_2^-$	1.0	−25.2	+10.9	(2.0±0.1) × 10^6	−36.1
$MeSO_3^-$	0.8	−42.1	−8.0	(9.4±1.0) × 10^5	−34.1
NO_3^-	0.9	−55.2	−1.0	(3.0±0.3) × 10^9	−54.2
ReO_4^-	1.0*	−52.5	−9.9	(3.0±0.9) × 10^7	−42.6
ClO_4^-	0.9	−68.9	−16.1	(1.7±0.1) × 10^9	−52.8
PF_6^-	0.9	−69.7	−20.5	(4.1±0.3) × 10^8	−49.2
F^-	1.0	−52.7	−7.6	(7.7±0.1) × 10^7	−45.1
Cl^-	0.9	−59.1	−9.8	(4.2±0.1) × 10^8	−49.3
Br^-	0.9	−71.7	−14.6	(9.8±0.1) × 10^9	−57.1
I^-	1.0	−77.6	−14.3	(1.2±0.1) × 10^{11}	−63.3

*The N value was fixed.

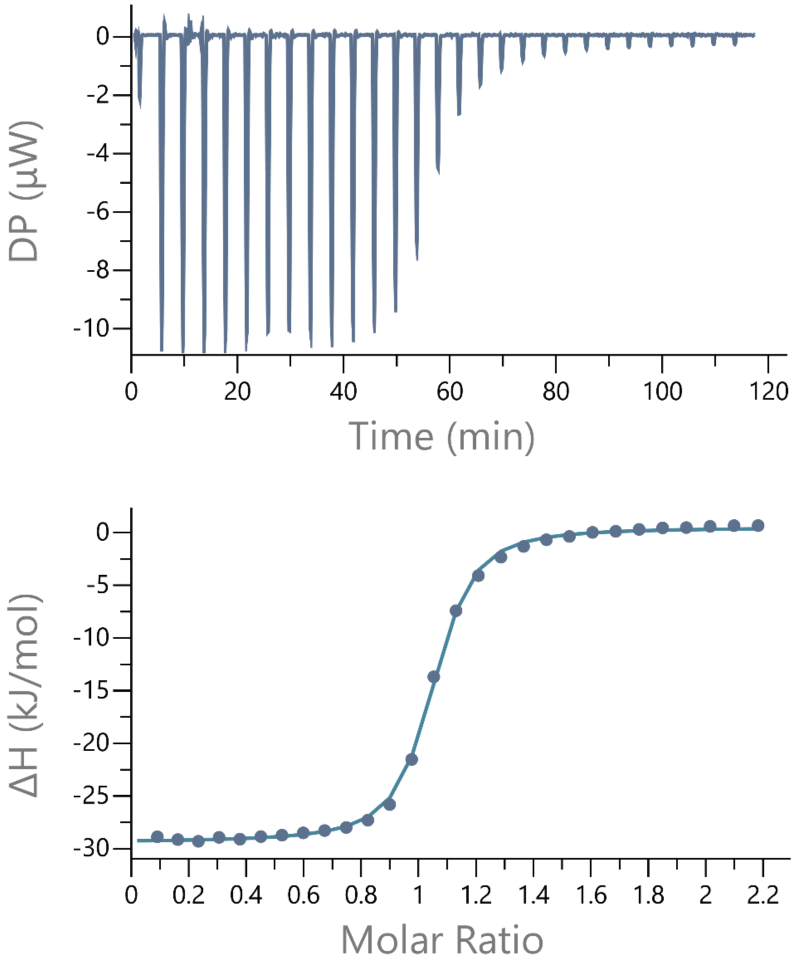

Figure S21. ITC titration of BU1 (0.075 mM) with tetrabutylammonium (TBA) nitrate (0.75 mM) in acetonitrile.

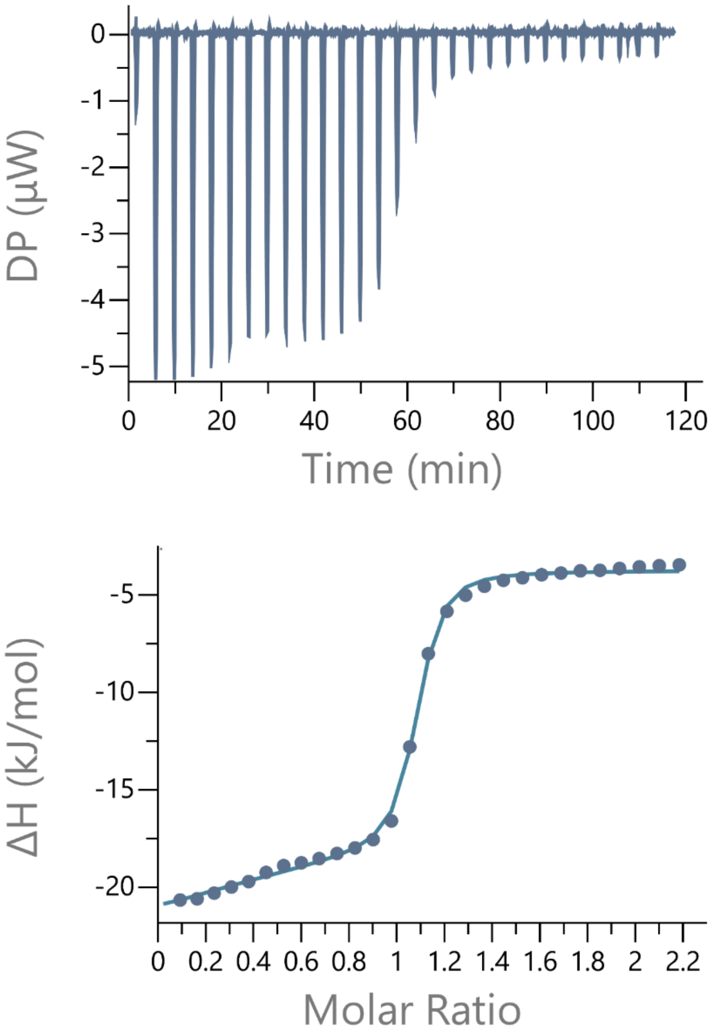

Figure S22. ITC titration of BU1 (0.062 mM) with tetrabutylammonium (TBA) bromide (0.62 mM) in the presence of $TBAMeSO_3$ (0.120 mM) in acetonitrile.

Table S3 Apparent association constants (K_a), binding stochiometry (N), and thermodynamical parameters (ΔH, $T\Delta S$, ΔG) of the tetrabutylammonium (TBA) anion⊂BU1 complexes determined by ITC in acetonitrile at 298.15 K

Anion	N	ΔH (kJ mol^{-1})	$T\Delta S$ (kJ mol^{-1})	K_a (M^{-1})	ΔG (kJ mol^{-1})
**NO_3^-**	1.0	–30.2	+6.8	(2.9±0.1) × 10^6	–37.0
**Br^-**	0.9	–28.6	+13.8	(2.6±0.1) × 10^7	–42.4

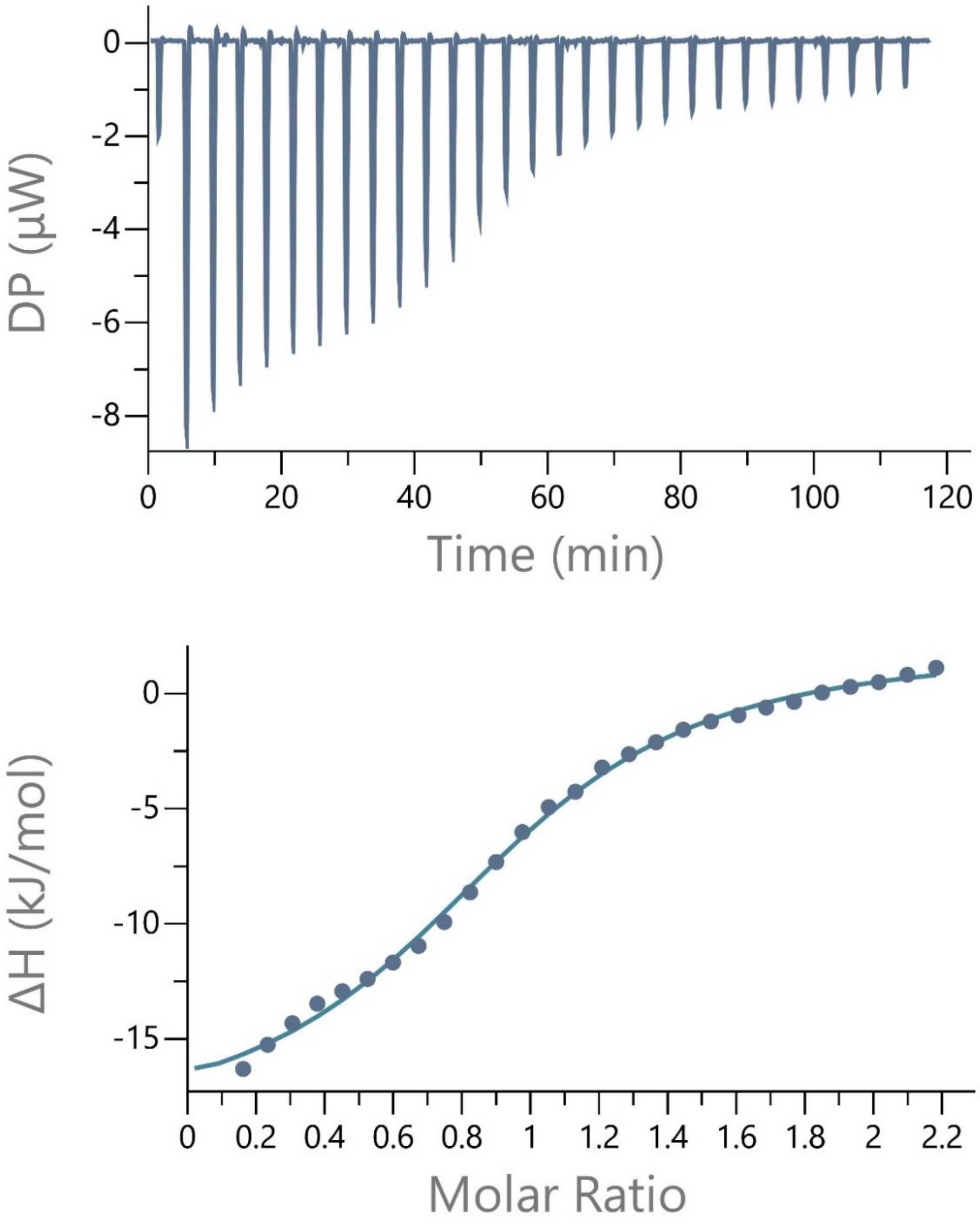

Figure S23. ITC titration of BU1 (0.1 mM) with tetraethylammonium (TEA) chloride (0.62 mM) in chloroform.

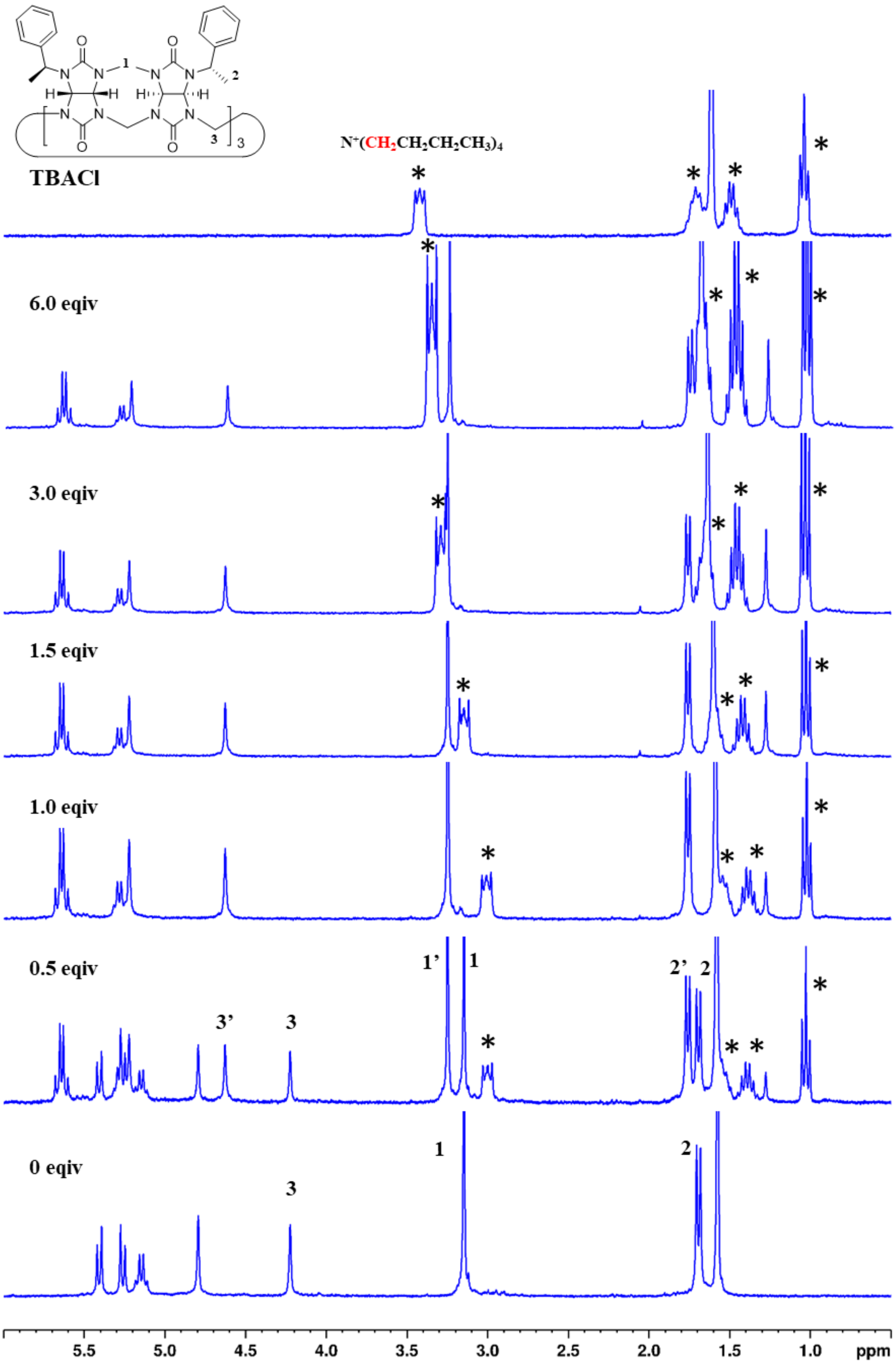

Figure S24. ^{1}H NMR (CDCl3, 300 MHz, 30 °C) stacked spectra of BU2 (1 mM) with increasing concentration of TBACl, and TBACl alone (the upper spetra). * TBA proton signals. Complete dissociation of ion pairs is indicated by an upfield shift of the TBA signal (N+CH2). The chemical shift remains constant below one equivalent of added salt.

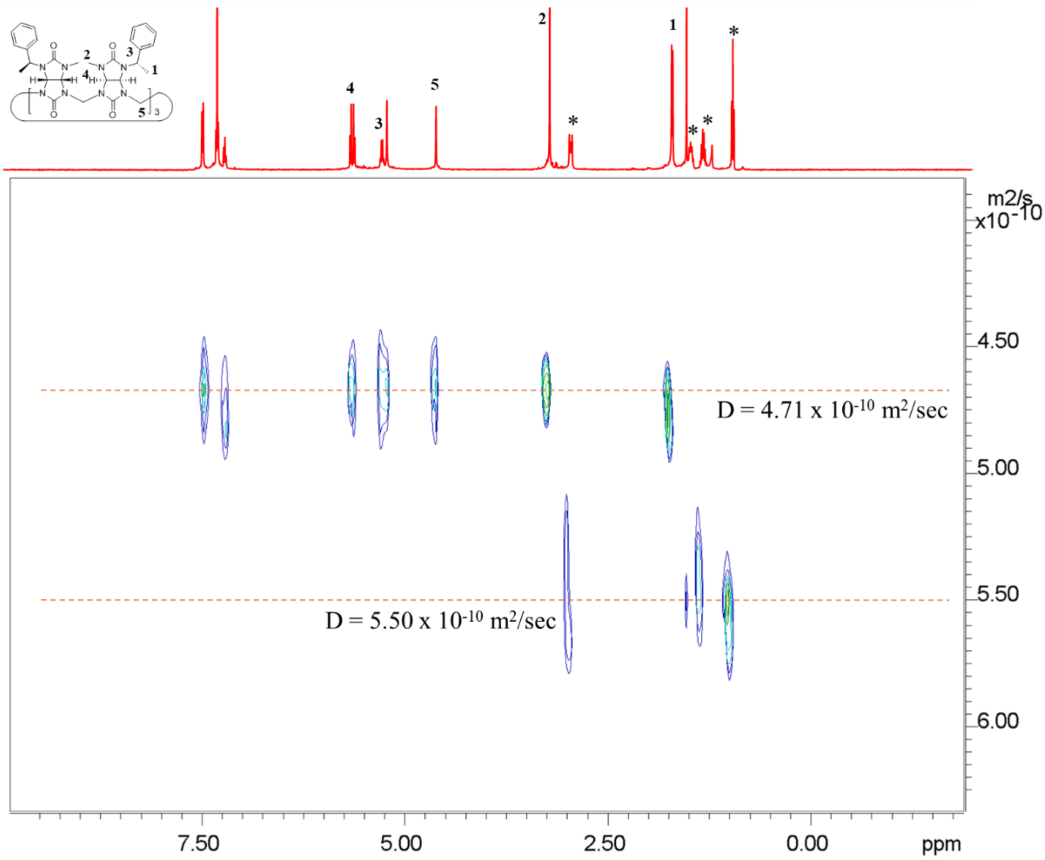

Figure S25. DOSY spectrum (500 MHz, $CDCl_3$, 298.15 K) of the equimolar mixture of TBACl and BU2 (1mM). * TBA proton signals.

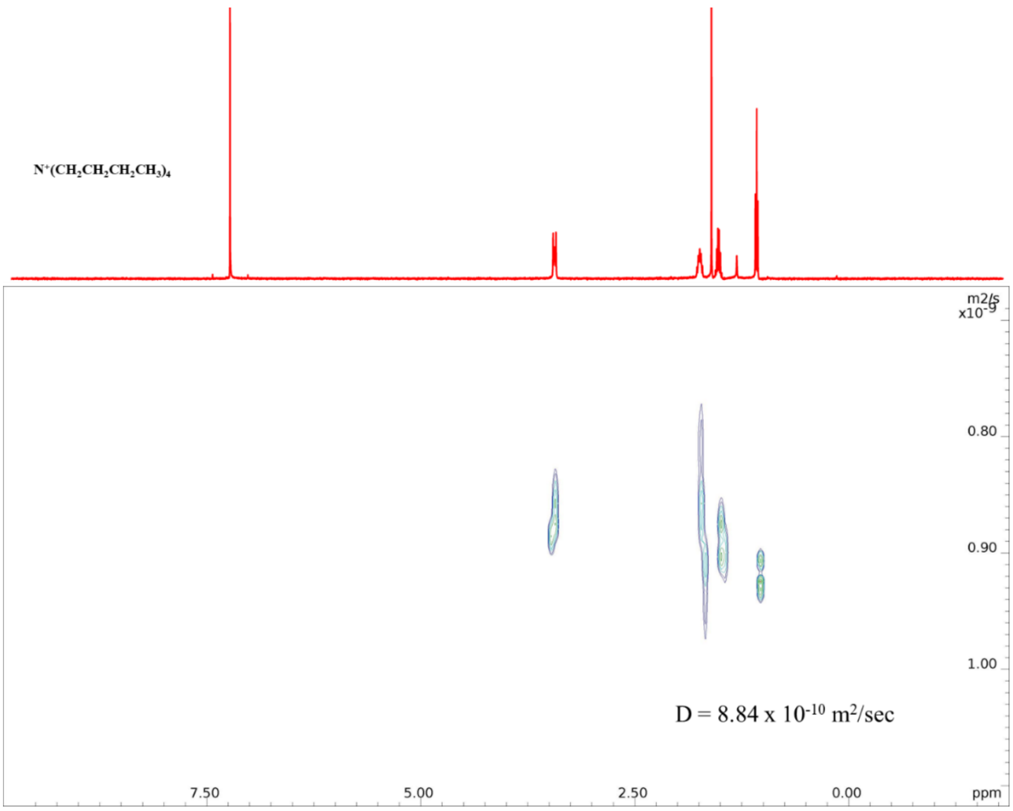

Figure S26. DOSY spectrum (500 MHz, $CDCl_3$, 298.15 K) of the TBACl^- (1mM).

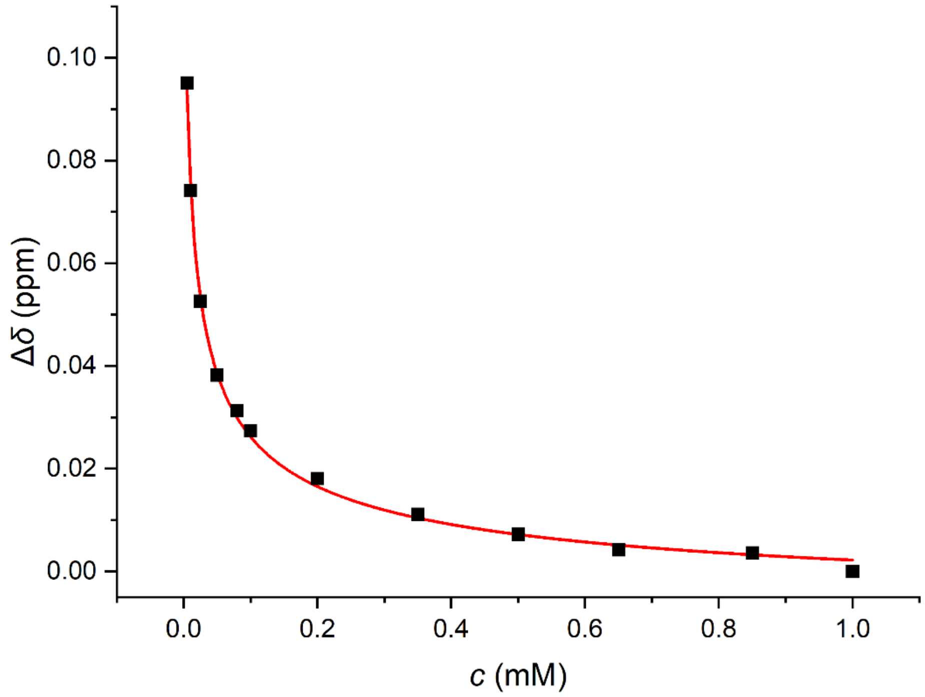

Figure S27. ^{1}H NMR (500 MHz, $CDCl_3$, 298.15 K) dilution experiment of equimolar mixture of TBACl and BU2 (0.005 – 1 mM). The peak shift of α-metylene protons of TBA^+ during variable-concentration ^{1}H NMR experiments. The experimental points of chemical shifts are showed as black squares. Red line shows fitted curve which yielded ion-pair association constant (K_{ip}) of (1.2 ± 0.2) × 10^5 M^{-1}.

Computational Methods

All quantum-chemical (QM) calculations were done in Orca v4.2.1.[3] We built all structures *in silico* and optimised them at the B97-3c level of theory[4] in a vacuum. Optimised structures were subject to single-point energy calculations at the PBE0-D3BJ (RIJCOSX) level of theory.[5–8]def2-TZVPP basis set was employed for all except halogen atoms.[9] Due to their anionic nature, the def2-TZVPPD basis set with diffuse functions had to be used. Moreover, the calculated interaction energies were corrected for BSSE errors employing the counterpoise method.[10] The effect of solvent (chloroform) was included using the SMD model.[11] The absolute energies of individual systems and their parts are summarised in Table S4. Optimised geometries of all systems are attached in a zip archive.

Table S4. Absolute energies of BU1 and BU2 in the free form and complexed with halide anions. E_{vac} is energy in a vacuum; E_{vac}/BU is the energy of macrocycle in the geometry taken from the complex; E_{vac}/[BU]X is the energy of the complex where macrocycle atoms are considered as ghost atoms; E_{vac}/BU[X] is the energy of the complex where halide atoms are considered as ghost atoms; E_{SMD} is energy in implicit chloroform solvent. All energies are in atomic units (Hartrees).

	closed conformation					open conformation				
	E_{vac}	E_{vac}/BU	E_{vac}/[BU]X	E_{vac}/BU[X]	E_{SMD}	E_{vac}	E_{vac}/BU	E_{vac}/[BU]X	E_{vac}/BU[X]	E_{SMD}
**BU1**	-5472.065260				-5472.180359	-5472.041110				-5472.159163
**BU1/F(-)**	-5571.982373	-5472.054597	-99.796610	-5472.054733	-5572.105068	-5571.964733	-5472.028719	-99.796611	-5472.028907	-5572.095569
**BU1/Cl(-)**	-5932.309605	-5472.057090	-460.129848	-5472.057386	-5932.428131	-5932.281259	-5472.033646	-460.129791	-5472.033935	-5932.410267
**BU1/Br(-)**	-8046.119151	-5472.058380	-2573.940260	-5472.058762	-8046.238045	-8046.088556	-5472.034075	-2573.940209	-5472.034476	-8046.219012
**BU1/I(-)**	-5769.989017	-5472.059760	-297.812571	-5472.060213	-5770.105072	-5769.956187	-5472.034582	-297.812559	-5472.035045	-5770.085961
	closed conformation					open conformation				
	E_{vac}	E_{vac}/BU	E_{vac}/[BU]X	E_{vac}/BU[X]	E_{SMD}	E_{vac}	E_{vac}/BU	E_{vac}/[BU]X	E_{vac}/BU[X]	E_{SMD}
**BU2**	-5472.045065				-5472.161239	-5472.033552				-5472.163686
**BU2/F(-)**	-5571.925922	-5472.034829	-99.796852	-5472.034924	-5572.057613	-5571.959636	-5472.019850	-99.796637	-5472.020027	-5572.095950
**BU2/Cl(-)**	-5932.264197	-5472.035536	-460.129800	-5472.035856	-5932.391414	-5932.287301	-5472.024763	-460.129766	-5472.025143	-5932.418632
**BU2/Br(-)**	-8046.076203	-5472.036021	-2573.940092	-5472.036295	-8046.196851	-8046.093626	-5472.025531	-2573.940140	-5472.025903	-8046.225256
**BU2/I(-)**	-5769.950711	-5472.036948	-297.812503	-5472.037382	-5770.066982	-5769.959943	-5472.026773	-297.812539	-5472.027161	-5770.090458

Results and Discussion

The 1-phenylethyl groups attached to the glycoluril moieties are rotatable. This flexibility results in various levels of macrocycle cavity shielding from the solvent. We tested two boundary conformations: open and closed (Figure 2 and Table S5). QM calculations indicate that **BU1** prefers the closed form, while **BU2** prefers the open form.

Table S5. The energy difference ΔE between closed and open forms in implicit chloroform solvent. A negative value indicates a preference for the closed form. All energies are in kJ/mol.

	ΔE_{SMD}(closed-open)	
	**BU1**	**BU2**
**BU**	-55.7	6.4
**BU/F(-)**	-24.9	100.7
**BU/Cl(-)**	-46.9	71.5
**BU/Br(-)**	-50.0	74.6
**BU/I(-)**	-50.2	61.6

Next, we determined the binding affinities of halide anions into the bambus[6]uril cavity. In agreement with the experimental measurements, **BU2** exhibits bigger binding affinities than **BU1** (Table 1). Further, we decomposed the obtained binding affinities into interaction, deformation, and solvation contributions to trace the origin of this preference (see the main text for details).

Detailed analysis of complexes revealed that **BU2** provides a more effective hydrogen bond network between methine hydrogen atoms with the bound halide anions than **BU1** (Table S6).

Table S6. Average distances between two methine hydrogen (H_a and H_b) atoms and halide anion. Δd is the difference between the two sites. All values are in Angstroms (Å).

	**BU1**				**BU2**		
	**<d(H_a-X)>**	**<d(H_b-X)>**	**Δd**		**<d(H_a-X)>**	**<d(H_b-X)>**	**Δd**
**BU/F(-)**	3.03	3.36	0.33		2.62	2.89	0.27
**BU/Cl(-)**	2.94	3.28	0.34		2.93	2.87	-0.06
**BU/Br(-)**	2.99	3.30	0.31		2.99	2.93	-0.05
**BU/I(-)**	3.06	3.32	0.26		3.12	3.07	-0.04

References

1 J. Sokolov and V. Šindelář, *Chem. - Eur. J.*, 2018, **24**, 15482–15485.
2 V. Havel and V. Sindelar, *ChemPlusChem*, 2015, **80**, 1601–1606.
3 F. Neese, *Wiley Interdiscip. Rev.-Comput. Mol. Sci.*, 2012, **2**, 73–78.
4 J. G. Brandenburg, C. Bannwarth, A. Hansen and S. Grimme, *J. Chem. Phys.*, 2018, **148**, 064104.
5 C. Adamo and V. Barone, *J. Chem. Phys.*, 1999, **110**, 6158–6170.
6 J. P. Perdew, K. Burke and M. Ernzerhof, *Phys. Rev. Lett.*, 1996, **77**, 3865–3868.
7 F. Neese, F. Wennmohs, A. Hansen and U. Becker, *Chem. Phys.*, 2009, **356**, 98–109.
8 S. Grimme, S. Ehrlich and L. Goerigk, *J. Comput. Chem.*, 2011, **32**, 1456–1465.
9 F. Weigend and R. Ahlrichs, *Phys. Chem. Chem. Phys.*, 2005, **7**, 3297–3305.
10 S. Boys and F. Bernardi, *Mol. Phys.*, 1970, **19**, 553-.
11 A. V. Marenich, C. J. Cramer and D. G. Truhlar, *J. Phys. Chem. B*, 2009, **113**, 6378–6396.
